# Membrane-active macromolecules kill antibiotic-tolerant bacteria and potentiate antibiotics towards Gram-negative bacteria

**DOI:** 10.1371/journal.pone.0183263

**Published:** 2017-08-24

**Authors:** Divakara S. S. M. Uppu, Mohini M. Konai, Paramita Sarkar, Sandip Samaddar, Isabel C. M. Fensterseifer, Celio Farias-Junior, Paramanandam Krishnamoorthy, Bibek R. Shome, Octávio L. Franco, Jayanta Haldar

**Affiliations:** 1 Chemical Biology & Medicinal Chemistry Laboratory, New Chemistry Unit, Jawaharlal Nehru Centre for Advanced Scientific Research (JNCASR), Bangalore, Karnataka, India; 2 Centro de Análises Proteômicas e Bioquímicas, Pós-Graduação em Ciências Genômicas e Biotecnologia UC, Brası´lia, Brazil; 3 Molecular Pathology Post-Graduate Program, University of Brasília, Brasília, Brazil; 4 Lacen, Laboratorio Central, Brasília, DF, Brazil; 5 ICAR-National Institute of Veterinary Epidemiology and Disease Informatics (NIVEDI), Bengaluru, Karnataka, India; 6 S-inova Biotech, Pos-Graduação em Biotecnoloia, Universidade Catolica Dom Bosco, Campo Grande, Brazil; Institut Pasteur, FRANCE

## Abstract

Chronic bacterial biofilms place a massive burden on healthcare due to the presence of antibiotic-tolerant dormant bacteria. Some of the conventional antibiotics such as erythromycin, vancomycin, linezolid, rifampicin etc. are inherently ineffective against Gram-negative bacteria, particularly in their biofilms. Here, we report membrane-active macromolecules that kill slow dividing stationary-phase and antibiotic tolerant cells of Gram-negative bacteria. More importantly, these molecules potentiate antibiotics (erythromycin and rifampicin) to biofilms of Gram-negative bacteria. These molecules eliminate planktonic bacteria that are liberated after dispersion of biofilms (dispersed cells). The membrane-active mechanism of these molecules forms the key for potentiating the established antibiotics. Further, we demonstrate that the combination of macromolecules and antibiotics significantly reduces bacterial burden in mouse burn and surgical wound infection models caused by *Acinetobacter baumannii* and Carbapenemase producing *Klebsiella pneumoniae* (KPC) clinical isolate respectively. Colistin, a well-known antibiotic targeting the lipopolysaccharide (LPS) of Gram-negative bacteria fails to kill antibiotic tolerant cells and dispersed cells (from biofilms) and bacteria develop resistance to it. On the contrary, these macromolecules prevent or delay the development of bacterial resistance to known antibiotics. Our findings emphasize the potential of targeting the bacterial membrane in antibiotic potentiation for disruption of biofilms and suggest a promising strategy towards developing therapies for topical treatment of Gram-negative infections.

## Introduction

Bacterial infections relapse even after the treatment with antibiotics [1, 2]. Bacterial biofilms are the root-cause of recurring infections as they are tolerant to antibiotic treatment and host immune system [1]. Biofilms contain bacteria and extracellular polymeric substances (EPS) [2]. It has been found that biofilms account for 80% of human bacterial infections [3] [4]. Tolerance of biofilms to conventional antibiotic treatment is due to slow or non-dividing cells, entry barriers and genetic changes. Moreover, antibiotic resistance worsens the situation of chronic and relapsing infections. Antibiotic tolerance is a phenomenon exhibited by whole population of bacteria to survive transient exposure to high concentration of antibiotics by slowing down essential bacterial process. On the other hand, persisters are a small sub-population of survivors post antibiotic treatment that play a major role in chronic biofilm infections [5–9]. Persistence and antibiotic tolerance are survival strategies employed by the bacteria and they revert to their normal growth conditions after the antibiotic treatment. These transient phenotypic variants (dormant) of bacteria after growth resumption act as a pool for the development of antibiotic-resistant bacteria and are the underlying cause for relapsing infections [9, 10]. Thus, targeting dormant persisters or antibiotic tolerant cells, the root-cause, might lead to curbing antibiotic resistance and preventing chronic biofilm infections.

Persisters and antibiotic tolerant cells with reduced metabolic activity exhibit antibiotic-tolerance because the bio-synthetic processes that are the targets for known antibiotics are either inactive or corrupt in these cells [8]. Thus, due to the presence of persisters, the target-specific mode of action of established antibiotics proved unsuccessful for the eradication of chronic infections [8]. Hence, there is a need for developing strategies for targeting persister and antibiotic cell populations. One such strategy is to target the bacterial cell membrane which is required for maintaining viability in their active as well as metabolically inactive (dormant) state [11]. Cationic and hydrophobic polymers are promising for targeting antibiotic tolerant bacteria, persisters and disrupting biofilms [12–22] as they have been known to interact with bacterial lipid membranes [23–28]. The membrane-disruptive nature of these polymers might target persister and antibiotic tolerant bacteria in biofilms. We hypothesized that these cationic and hydrophobic macromolecules might also efficiently interact with the negatively charged EPS matrix thus weakening the biofilms. Membrane-targeting compounds are also interesting due to their low tendency for the development of bacterial resistance [23].

**Fig 1:**
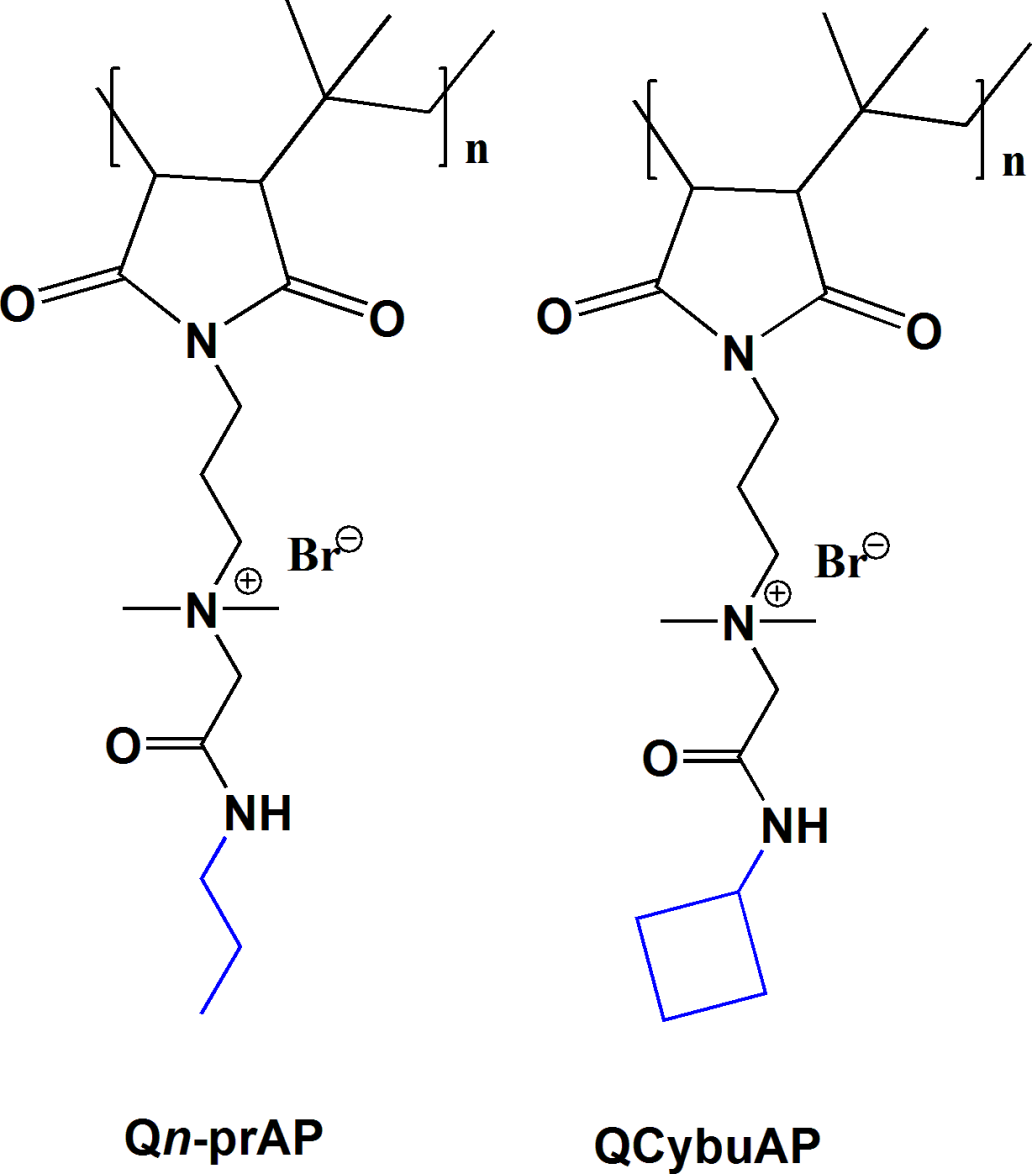
Chemical structures of the membrane-active macromolecules.

Here, we report amphiphilic macromolecules that potentiate conventional antibiotics to disrupt established Gram-negative biofilms on surfaces (Fig 1). The antibacterial activity against slow or non-dividing cells such as stationary phase and antibiotic-tolerant bacteria is determined along with their membrane-active mechanism. More importantly, the molecules in combination with known antibiotics show excellent efficacy in mouse burn and surgical wound infection models caused by *A*. *baumannii* and *K*. *pneumoniae* (KPC) clinical isolate respectively. Interestingly, these molecules delay the development of bacterial resistance to existing antibiotics in Gram-negative bacteria.

## Materials and methods

### Ethics statement

Animal studies were performed according to the protocols approved by Institutional Animal Ethics Committee (IAEC) of JNCASR and ICAR-NIVEDI. Toxicity studies were performed at JNCASR, Bengaluru (CPCSEA/201) and infection studies were performed at NIVEDI approved by the IAEC of NIVEDI, Bengaluru (881/GO/ac/05/CPCSEA) and the Catholic University of Brasilia (number 005/13). Animal handling, experimentation protocols, use of humane endpoints, euthanization procedures (using Isoflurane (Halothane) inhalant anaesthetic or carbon dioxide asphyxiation) were performed as per the OECD guidelines and adhere to the ARRIVE guidelines (S1 Checklist).

### Compounds

Synthesis and characterization of cationic amphiphilic macromolecules was as described previously [29–32]. Briefly, to a solution of 0.5 g of *Poly(isobutylene-alt-N-(N'*,*N'-dimethylaminopropyl)-maleimide)* (PIBMI) [29–32] in 20 mL of dry DMF/dry CHCl_3_ (1:1), 2 equivalents (with respect to the monomer weight of PIBMI) of *N*-alkyl-1-bromoethanamide [29–32] was added and stirred at 75°C for 96 h in a screw top pressure tube. The solution was cooled, precipitated with 40 mL of diethyl ether and filtered. The white solid was washed with diethyl ether (4 × 40 mL) and dried at 40°C for 6 h under vacuum. The characterization of the compounds has been described previously [28–31].

### Bacterial strains, culture media and antibiotics

Optical density and absorbance were measured by Tecan InfinitePro series M200 Microplate Reader. Bacterial strains *E*. *coli* (MTCC 443 equivalent to ATCC 25922) and *A*. *baumannii* (MTCC 1425) were purchased from MTCC (Chandigarh, India). *E*. *coli* was cultured in Luria Bertani broth (10 g of tryptone, 5 g of yeast extract, and 10 g of NaCl in 1000 mL of sterile distilled water. *A*. *baumannii* were grown in nutrient broth (1 g of beef extract, 2 g of yeast extract, 5 g of peptone and 5 g of NaCl in 1000 mL of sterile distilled water). For solid media, 5% agar was used along with above mentioned composition. The bacterial samples were freeze dried and stored at -80°C. 5 μL of these stocks were added to 3 mL of the nutrient broth and the culture was grown for 6 h at 37°C prior to the experiments. *A*. *baumannii* R674 was from Department of Neuromicrobiology, National Institute of Mental Health and Neuro Sciences (NIMHANS), Hosur Road, Bangalore 560029, India. Clinical isolate, *K*. *pneumoniae* 003259271 (Hodge test positive, KPC positive and NDM-1 negative) was from Lacen, Laboratorio Central, Brasília, DF, Brazil. Culture media and all the antibiotics were from HIMEDIA and Sigma-Aldrich (India) respectively.

### *In-vitro* studies

#### Antibacterial activity against antibiotic-tolerant and stationary phase bacteria

Antibiotic-tolerant cells were generated by following a literature procedure [33]. 5 μL of the -80°C stock was added to 3 mL of the culture medium and grown for 6 h at 37°C. Then the bacterial suspension was diluted to 1:1000 and grown for 16 h to reach stationary phase (~10^9−10^ CFU mL^-1^). 1 ml of this bacterial suspension was treated with 300 μg mL^-1^ (*E*. *coli*) of ampicillin sodium for 3 h at 37°C. After 3h, the bacteria were centrifuged, washed three-four times and resuspended in M9 media (*E*. *coli*) to remove the traces of the antibiotic. Then the bacterial suspension was diluted and spot-plated on LB agar (*E*. *coli*) plates for determining the count of antibiotic-tolerant cells. An antibiotic-tolerant cell count of ~10^8−9^ CFU mL^-1^ was obtained stating the difference between stationary phase and antibiotic-tolerant populations (S1 Fig). To confirm the tolerance stage even more clearly, we further treated the above obtained antibiotic-tolerant cells with ampicillin sodium for 2 h and we found no reduction in bacterial counts.

Both the stationary phase and antibiotic-tolerant cells were diluted finally to ~10^6^ CFU mL^-1^ in M9 media (*E*. *coli*). For *A*. *baumannii* bacteria were grown to stationary phase in LB media as described above for 16 h to obtain ~10^10^ CFU mL^-1^ and were finally diluted to ~10^6^ CFU mL^-1^ in BM2 media ((NH_4_)_2_SO_4_ and potassium phosphate buffer, pH = 7, 0.5% glucose) supplemented with 200 μM FeCl_3_ and 0.5% casamino acids. 50 μL of compounds (antibiotics or macromolecules) were added to a 96 well plate (Polystyrene) containing 150 μL bacterial suspension. The plate was then incubated at 37°C for 2 h. After 2 h, 20 μL of the bacterial suspension in the well plate was serially diluted (10 fold) and spot-plated (20 μL) on agar plates. The viable colonies (<100) were counted after 48 h incubation at 37°C.

#### Antibacterial activity against actively growing bacteria in chemically defined media

5 μL of the -80°C stock was added to 3 mL of the broth and the culture was grown for 6 h at 37°C prior to the experiments contained ~10^8^ CFU mL^-1^. Actively growing cells (mid-log phase) were diluted to ~5×10^6^ CFU mL^-1^ in M9 media (*E*. *coli*) and to ~10^7^ CFU mL^-1^ in BM2 media ((NH_4_)_2_SO_4_ and potassium phosphate buffer, pH = 7, 0.5% glucose) supplemented with 200 μM FeCl_3_ and 0.5% casamino acids (*A*. *baumannii*). 50 μL of compounds (antibiotics or macromolecules) were added to a 96 well plate (Polystyrene) containing 150 μL bacterial solutions. The plate was then incubated at 37°C for a period of 2 h. After 2 h, 20 μL of the bacterial suspension was serially diluted (10 fold) and spot-plated (20 μL) on agar plates. The viable colonies (<100) were counted after 48 h incubation at 37°C.

#### Cytoplasmic membrane depolarization

Bacteria were harvested, washed with 5 mM HEPES and 5 mM glucose and resuspended in 5 mM glucose, 5 mM HEPES buffer and 100 mM KCl solution in 1:1:1 ratio (~10^8−9^ CFU mL^-1^). Measurements were made in a Corning 96 well black plate with clear bottom with 150 μl of bacterial suspension and 2 μM of DiSC_3_(5). 0.2 mM of EDTA was used to permeabilize the outer membrane and allow the dye uptake. The fluorescence of the dye was monitored using a Tecan InfinitePro series M200 Micro Plate Reader at excitation wavelength of 622 nm and emission wavelength of 670 nm. Dye uptake, and resultant self-quenching, was modulated by the membrane potential. After reaching the maximum uptake of the dye by bacteria, which was indicated by a minimum in dye fluorescence, polymer solution was added to the cells, and the decrease in potential was monitored by increase in fluorescence. All the other test compounds were dissolved in water at 4 mg mL^-1^ and DiSC_3_(5) dissolved in DMSO were further diluted in the above 5 mM glucose, 5 mM HEPES buffer and 100 mM KCl solution in 1:1:1 ratio. A control without the polymers was served as negative control.

#### Cytoplasmic membrane permeabilization

Bacteria were harvested, washed, and resuspended in 5 mM HEPES and 5 mM glucose buffer of pH 7.2 (~10^8−9^ CFU mL^-1^). Then, 150 μl of bacterial suspension, 10 μM propidium iodide (PI) and polymer solution were added to the cells in a Corning 96 well black plate with clear bottom. Stock solutions of PI and the polymers were made in water and further diluted in HEPES. Excitation wavelength of 535 nm and emission wavelength of 617 nm were used. The uptake of PI was measured using a Tecan InfinitePro series M200 Microplate Reader by the increase in fluorescence of PI for 30 min as a measure of membrane permeabilization.

#### Biofilm disruption assays [30]

#### Crystal violet staining of biofilms

Sterilized cover slips (diameter 13 mm) were placed in wells of a 6-well plate. 10^5^ CFU mL^-1^ of *E*. *coli* in M9 media supplemented with 0.5% glycerol and 0.02% Casamino acids. *A*. *baumannii* was diluted to approximately 10^5^ CFU mL^-1^ in BM2 media ((NH_4_)_2_SO_4_ and potassium phosphate buffer, pH = 7, 0.5% glucose) supplemented with 200 μM FeCl_3_ and 0.5% casamino acids. 2 mL of this bacterial suspension was added to wells containing the cover slips. The plate was incubated under stationary conditions at 30°C for 48 h (*A*. *baumannii*) and 72 h (*E*. *coli*). Afterwards media was removed and planktonic bacteria were carefully washed with 1X PBS (pH = 7.4) and removed. Biofilm containing cover slips were then placed into another 6-well plate and 2 mL of test compounds were added to it and allowed to incubate for 24 h. In case of control, 2 mL of media was added instead of compounds. At the end of 24 h, medium was then removed and planktonic cells were removed by washing with 1X PBS. The cover slips were carefully removed from the well and placed into another 6-well plate. For visualizing the disruption of biofilm, 100 μL of 0.1% of crystal violet (CV) was added into the wells and incubated for 10 min. After washing the excess dye, the glass cover slips stained with CV were dissolved in 95% ethanol and transferred to a fresh 96-well plate that were imaged with a digital camera.

#### Bacterial count enumeration in biofilms

As described above, at the end of 24 h of treatment, medium was then removed and planktonic cells were removed by washing with 1X PBS. The cover slips were carefully removed from the well and placed into another 12- well plate and treated with 0.05% trypsin-EDTA solution for dissolution of the biofilms for 20 min at 30°C. Cell suspension of biofilms was then assessed by plating serial 10-fold dilutions on agar plates. After 24 h of incubation, bacterial colonies were counted.

#### Confocal laser scanning microscopy (CLSM) of biofilms

As described above, the cover slips after 24 h treatment with the test compounds (including the untreated conditions) were carefully removed from the well and placed on glass slides. The biofilms were stained with 10 μL SYTO-9 (3 μM) and imaged using a Zeiss 510 Meta confocal laser-scanning microscope. The images were prepared using LSM 5 Image examiner and z-stack images were obtained using ImageJ software.

#### Analysis of dispersed cells in biofilms

Bacterial suspension in the surrounding media of the biofilm containing cover slip in the well plates after 24 h treatment from above experiments was collected. The O.D._600_ of this bacterial suspension was measured and 20 μL of this suspension was also spot plated on agar plates to see the viable bacteria.

#### Chequer board assays [34]

The combination antibacterial efficacy of macromolecules and antibiotics was measured in nutrient broth using chequer board assays in the following manner. A solution of 25 μL each of antibiotic and macromolecule was added into each well of a 96 well plate followed by 150 μL of bacterial suspension (~5.0 × 10^5^ CFU mL^-1^) and incubated at 37°C for 24 h. Bacterial suspension alone and nutrient broth alone served as controls. Each MIC was a result of two independent experiments.

#### Drug resistance study

In brief, the first MIC determination of macromolecule, antibiotics and macromolecule + antibiotics was performed as described above (Chequer board assays). Bacteria from triplicate wells at the concentration of one-half MIC were removed and used to prepare the bacterial dilution (~5.0×10^5^ CFU mL^-1^) for the next experiment. These bacterial suspensions were then used to perform the antibacterial assay that was repeated for 32 passages. The fold of increase in MIC was determined by normalizing the MIC at passage n to that of the first passage (MIC_n_/MIC_1_).

### Animal studies

The mice were housed in individually ventilated cages (IVC) maintained with controlled environment as per the standards. They are housing–pathogen free conventional caging system, bedding material–Corn Cob. The husbandry conditions: -Light: dark cycle- 12:12 hours, Animal Room Temp: 22 ± 2°C, Relative humidity: 30–40%, Access to feed and water: *ad libitum* and Water: RO Water. Animals were randomly selected, marked to permit individual identification and kept in their cages for at least 5 days before the experiment to allow for acclimatization to the experimental conditions. Animal handling and experimentation protocols were followed according to OECD Guidelines for the Testing of Chemicals (OECD 425). All care was taken to cause no pain to the animals. Humane endpoints were used to avoid unnecessary distress and suffering in animals following an experimental intervention that would lead to death. All sections of this report adhere to the ARRIVE Guidelines for reporting animal research. A completed ARRIVE guidelines checklist is included in S1 Checklist.

#### *In-vivo* toxicity

The experimentation protocols for the determination of dosage, number of animals per groups etc. were followed according to the OECD Guidelines for the Testing of Chemicals (OECD 425). Female Balb/c mice (6–8 weeks, 18–22 g) were used for systemic toxicity studies. Mice were randomized into control and test groups with 5 mice per group. Control groups received 200 μL of sterilized PBS (pH = 7.4). Different doses (5.5, 17.5, 55 and 175 mg kg^-1^) of the test drugs were used as per the OECD guidelines. 200 μL of the test drug solution in sterilized PBS (pH = 7.4) was injected into each mouse (5 mice per group) through intravenous (i.v.) (tail vein) route of administration. The mice in the high dose group (175 mg kg^-1^) immediately post-injection of the drug, showed clinical signs of tremors, recumbency, sever distress and convulsions, which were indicative of the impending death or moribund condition. Therefore, some which were in moribund condition were humanely euthanized using Isoflurane (Halothane) inhalant anaesthetic. For the intraperitoneal (i.p.) and subcutaneous (s.c.) (over the flank) routes of administration, the high dose (175 mg kg^-1^) was not injected to reduce the animal lethality. All the mice were monitored for 14 days post-treatment. Different routes of administration were used to find out the best method of administration of test drugs with minimal pain or lethality to the animals. The animals were closely monitored for the first 30 min to 4 hr for the first 24 hours of the initiation of the experiment. And then onwards, they were monitored daily for 14 days. During the observation period of 14 days, no onset of abnormality was found. The 50% lethal dose (LD_50_) was estimated according to the up- and -down method [35]. The remaining animals, post-experimentation, were euthanized using the same procedure.

#### *A*. *baumannii* burn wound infection [36]

Female Balb/c mice (6–8 weeks, 22–25 g) were anesthetized (n = 4 per group) with ketamine-xylazine cocktail, their dorsal surface shaved and cleansed. Six mm diameter burn wounds were created by applying a 120 s- heated brass bar for 10 s. Immediately after injury, burn wounds were infected with a mid-log phase bacterial inoculum of ~10^7^ CFU (20 μL from 10^9^ CFU/mL) of *A*. *baumannii* (MTCC 1425) prepared in PBS. Burn wounds were treated 3 h post infection and thereafter every 24 h for 5 days. Rifampicin stock solution (100 mg/ml) was prepared in DMSO and further diluted in sterile PBS (pH = 7.4). QCybuAP was dissolved in PBS. Burn wounds were treated with 40 μL of solutions containing rifampicin (5 mg kg^-1^), QCybuAP (50 mg kg^-1^), rifampicin + QCybuAP (5mg kg^-1^+ 50 mg kg^-1^) whereas 5% DMSO was used as untreated control. QCybuAP was dissolved in PBS. Erythromycin stock solution (100 mg/ml) was prepared in DMSO and further diluted in sterile PBS (pH = 7.4). Polymers were dissolved in sterile PBS (pH = 7.4). Burn wounds were treated with 40 μL of solutions containing Q*n*-prAP (50 mg kg^-1^), QCybuAP (50 mg kg^-1^), erythromycin (20 mg kg^-1^), erythromycin + Q*n*-prAP (20 mg kg^-1^ + 50 mg kg^-1^), erythromycin + QCybuAP (20 mg kg^-1^ + 50 mg kg^-1^) and colistin (5 mg kg^-1^). Mice were euthanized 6 days post-injury; the wounded muscle tissue was excised, weighed, and homogenized in 10 mL of PBS. Serial homogenate dilutions were plated on MacConkey agar (Himedia, India) and the results were stated as log (CFU g^-1^) of tissue. P value was calculated using one-way ANOVA (Dunnett's Multiple Comparison Test) between the control group and treatment groups and a value of P < 0.05 was considered significant.

#### *K*. *pneumoniae* murine surgical wound infection

Female C57BL/6 mice (8–10 weeks old) were obtained from animal facility of the Catholic University of Brasilia. The murine surgical wound infection model was performed as described previously [37] with minor modifications. Mice (n = 4 per group) were anesthetized, their dorsal surface shaved and a puncture was performed using 6-mm punch biopsy needles (Stiefel Laboratories, UK) and then 20 μL of clinical isolate of tetracycline-resistant *K*. *pneumoniae-*003259271 (carbapenemase producing strain, KPC) suspension (2×10^9^ CFU mL^-1^) was introduced into the puncture wound. Wounds were treated every 24 h with 20 μL of a solution containing 50 mg kg^-1^ of Q*n*-prAP, 100 mg kg^-1^ of tetracycline, 5 mg kg^-1^ of colistin, 100 mg kg^-1^ + 50 mg kg^-1^ of tetracycline + Q*n*-prAP and a group not treated served as negative control. Mice were euthanized 7 days post-surgery and the wounded tissue was excised, weighed, and homogenized in 1 mL of PBS. Serial homogenate dilutions were plated in triplicate on Muller Hinton agar (Himedia, India) and the results were stated as log (CFU g^-1^) of tissue. P value was calculated using one-way ANOVA (Dunnett's Multiple Comparison Test) between the control group and treatment groups and a value of P < 0.05 was considered significant.

#### Histopathology

The skin tissue collected and fixed in 10% formalin for 48 hr and washed for 1 h in water. Dehydration of the tissues was performed with increasing concentrations of ethanol (70, 90 and 100%; each for 1 h) and cleared in xylene for 1 h twice. After paraffin embedding in melted paraffin at 56°C thrice, longitudinal and transverse sections (5 μm) were prepared with semiautomatic microtome and placed on glass slide coated with Meyer’s egg albumin. Tissue sections were dried by incubating them for 2 h at 40°C and rehydration of fixed sections was carried in decreasing grades of alcohol (100%, 90%, 70% and 50%; each for 1 h) and then water. Sections were stained with haematoxylin and eosin stain and covered with DPX (SRL, India) mounting medium with cover glass and observed under light microscope (Nikon, Japan) to study the histopathological changes.

## Results and discussion

### Cationic-amphiphilic macromolecules

We have reported the synthesis and characterization of cationic-amphiphilic macromolecules based on poly(isobutylene-*alt*-*N*-alkyl maleimide) backbone previously [30–32]. The detailed synthetic protocols and complete characterization have been reported previously [30–32]. These macromolecules selectively displayed potent antibacterial efficacy against actively growing planktonic bacteria with minimal toxicity to mammalian cells with membrane-active mode of action as described earlier [30–32]. From these previously published reports, we obtained two optimized macromolecules (shown in Fig 1) with high *in-vitro* selectivity for killing the bacteria sparing the mammalian cells [30–32]. Also, these two macromolecules alone were previously reported to disrupt biofilms and were effective towards chronic infections of *A*. *baumannii* in mice models [30]. In the present report, we aim to test the activity of these molecules against stationary phase as well as antibiotic-tolerant cells, dispersed cells, their ability to potentiate the conventional antibiotics against biofilms, in various mouse models of burn/surgical wound infections and to delay the development of bacterial resistance to old antibiotics.

### Antibacterial activity against planktonic bacteria

#### Antibacterial activity against actively growing bacteria

A detailed structure-activity relationship study from our previous reports have resulted in two optimized membrane-active macromolecules (Fig 1) with potent antibacterial activity and minimal mammalian toxicity. These molecules showed impressive selectivity (ratio of MIC (minimum inhibitory concentration) and HC_50_ (concentration at which 50% hemolysis of human red blood cells)) of 50–250 fold of killing bacteria compared to mammalian cells [30–32]. This led us to choose these two best macromolecules for further studies in this manuscript.

#### Antibacterial activity against dormant bacteria

**Fig 2:**
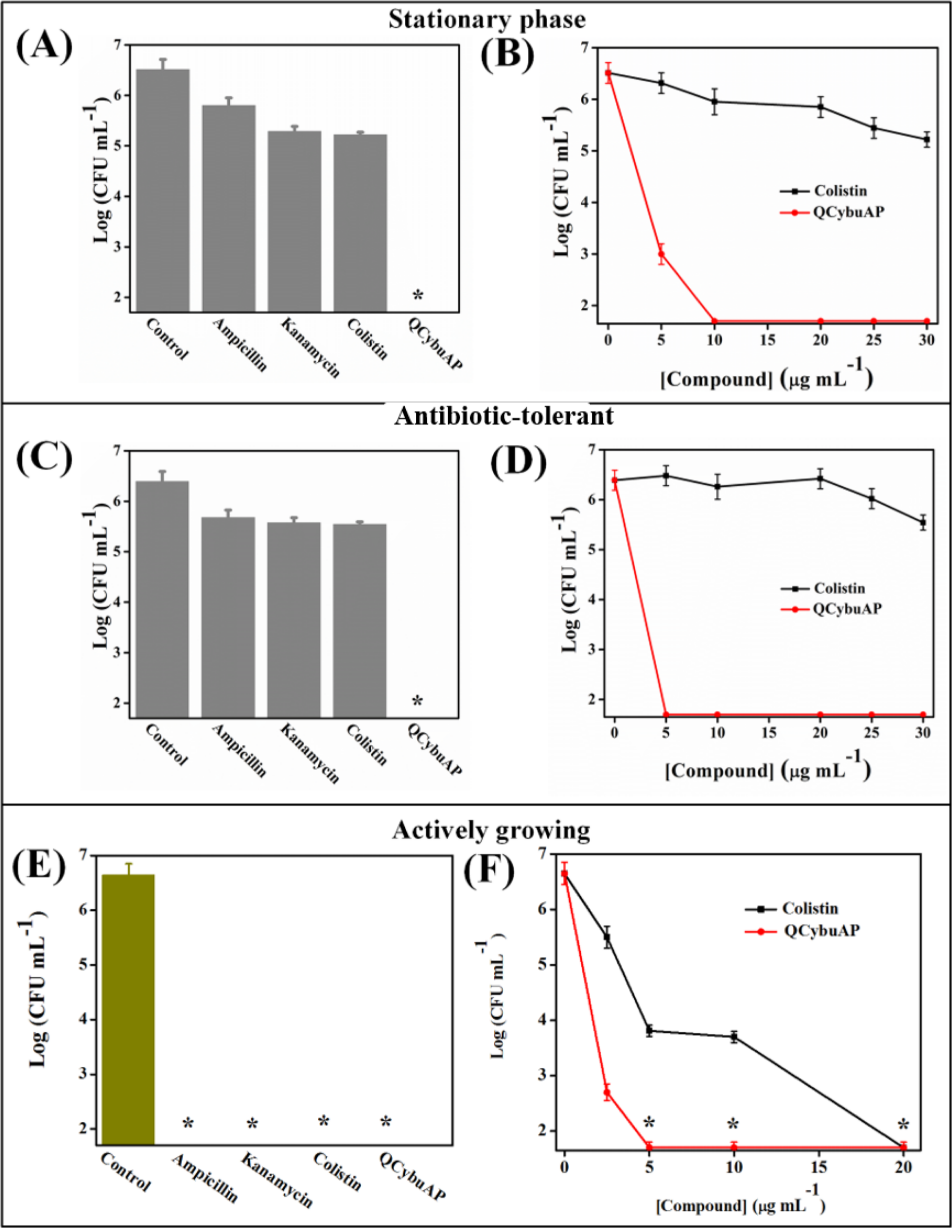
Antibacterial activity of antibiotics and QCybuAP against *E*. *coli*. ~10^6^ CFU mL^-1^ of bacteria in M9 media were treated with compounds for 2 h. Stars represent no survival detected (limit of detection < 50 CFU/mL).

We investigated the antibacterial efficacy of these cationic amphiphilic macromolecules against stationary phase and antibiotic-tolerant cells. The antibacterial activity was evaluated by determining the reduction in bacterial counts of actively growing, stationary phase and antibiotic-tolerant cells after 2 h treatment in chemically defined media, M9 complete medium (*E*. *coli*) [38, 39]. Bacteria were grown to stationary phase by culturing for 16 h at 37°C and diluted in fresh media to a final concentration of ~10^6^ CFU mL^-1^ for treatment with various antibacterial agents as described in Materials and Methods. Antibiotics such as ampicillin and kanamycin at 100 μg mL^-1^ and the membrane active drug, colistin, even at 30 μg mL^-1^ showed little bactericidal activity against stationary phase *E*. *coli* cells (Fig 2A). QCybuAP showed concentration dependent reduction in bacterial counts against stationary phase *E*. *coli* with complete killing at 10 μg mL^-1^ (Fig 2B).

**Fig 3:**
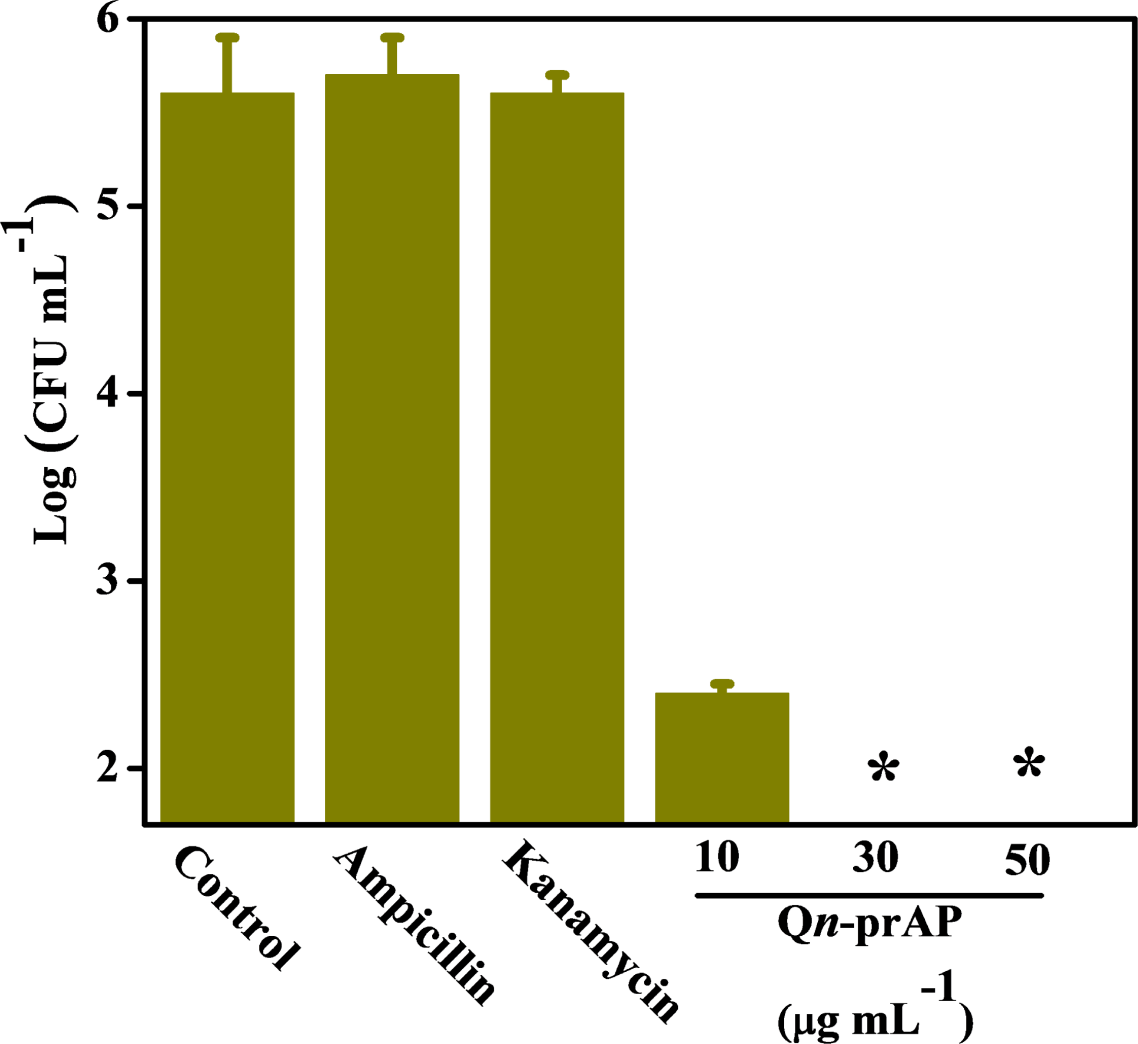
Antibacterial activity of antibiotics and cationic polymer (Q*n*-prAP) against *E*. *coli* antibiotic-tolerant cells. (A) Ampicillin (100 μg mL^-1^), kanamycin (100 μg mL^-1^) do not kill whereas Q*n*-prAP shows concentration dependent (10, 30 and 50 μg mL^-1^) elimination of *E*. *coli* antibiotic-tolerant cells. ~10^6^ CFU mL^-1^ of bacteria in M9 media were treated with compounds for 2 h. Stars represent no survival detected (limit of detection < 50 CFU/mL).

**Fig 4:**
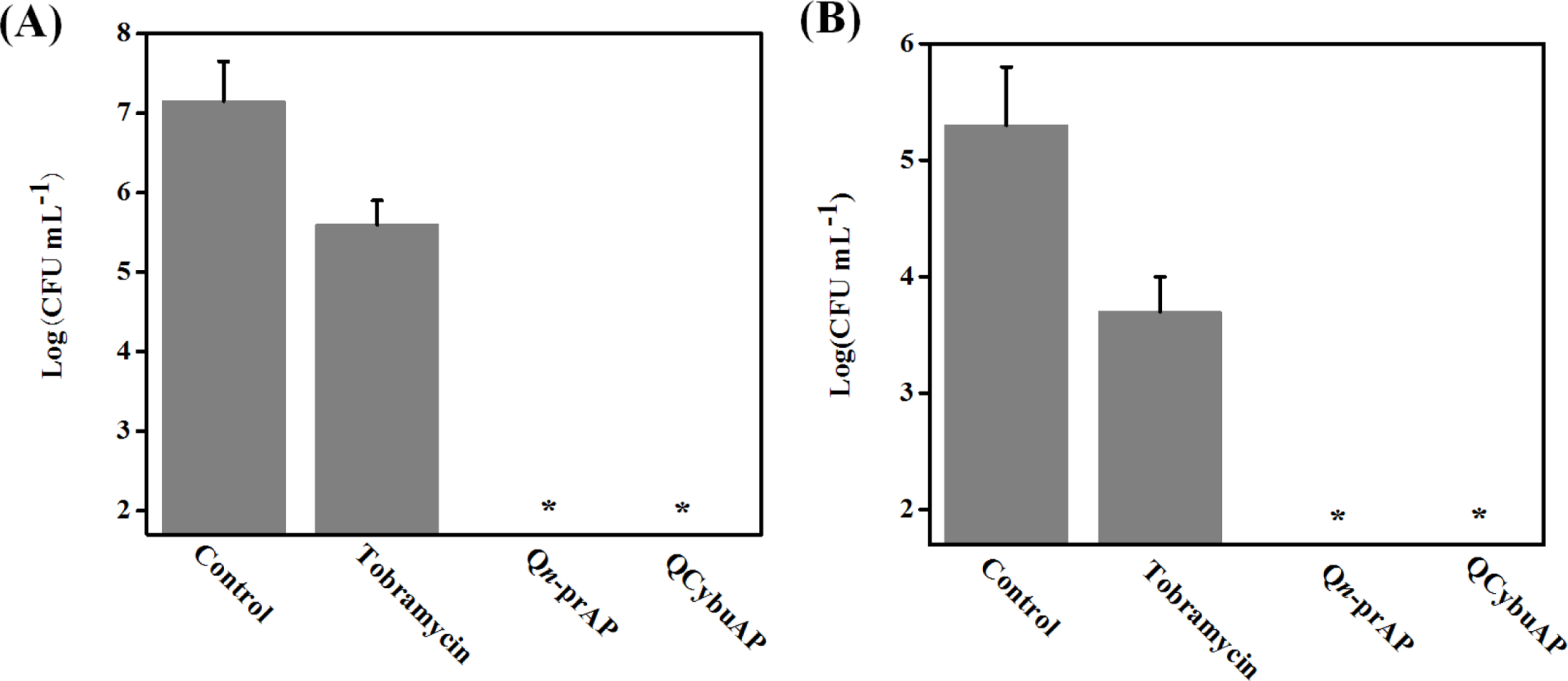
Antibacterial activity of antibiotics and cationic polymers (Q*n*-prAP) against *A*. *baumannii*. (A) Actively growing and (B) stationary phase cells. ~10^6^ CFU mL^-1^ of bacteria in BM2 media were treated with compounds for 2 h at a concentration of 10 μg mL^-1^. Stars represent no survival detected (limit of detection < 50 CFU/mL).

Antibiotic-tolerant cells were isolated by treating the stationary phase cultures of *E*. *coli* with ampicillin as described in the Methods section [38, 39]. The surviving antibiotic-tolerant cells of *E*. *coli* were isolated and used for the activity studies. The tolerance stage was confirmed by further treating the surviving antibiotic-tolerant cells with ampicillin with no observed reduction in bacterial counts. The surviving antibiotic-tolerant cells were diluted to a final concentration of ~10^6^ CFU mL^-1^ for treatment with various antibacterial agents. QCybuAP showed concentration dependent reduction in bacterial count with complete elimination of bacteria at 5 μg mL^-1^ whereas ampicillin (100 μg mL^-1^), kanamycin (100 μg mL^-1^) and colistin (even at 30 μg mL^-1^) were less active against *E*. *coli* antibiotic-tolerant cells (Fig 2C & 2D). Q*n*-prAP also showed concentration dependent activity against *E*. *coli* antibiotic-tolerant cells and eradicates them at 30 μg mL^-1^ (Fig 3). This suggests the rapid killing ability of QCybuAP at lower concentrations (5 μg mL^-1^) compared to Q*n*-prAP (30 μg mL^-1^). Even against actively growing and stationary phase cells of *A*. *baumannii*, both the polymers showed complete killing whereas tobramycin was ineffective at 10 μg mL^-1^ (Fig 4). However, all the antibiotics (ampicillin, kanamycin and colistin) and QCybuAP were highly effective against the actively growing cells of *E*. *coli* (Fig 2E & 2F). These results suggest that the conventional antibiotics are ineffective against the metabolically inactive antibiotic-tolerant cells. Loss of efficacy by antibiotics such as ampicillin (β-lactam) and kanamycin (aminoglycoside) against the antibiotic-tolerant cells suggests the dormant and metabolically in-active state of these cells. Interestingly, colistin (Polymixin E), the Gram-negative membrane (lipopolysaccharide (LPS)) targeting antibiotic was also ineffective against stationary phase and antibiotic-tolerant cells of *E*. *coli* at a concentration of 30 μg mL^-1^. Polymixin B was reported to have reduced activity (200–1500 fold increase in MIC) towards stationary phase *S*. *typhimirium* [40]. It was also reported that the stationary phase cells of *A*. *baumannii* have reduced surface charge and more hydrophobicity compared to wild-type cells that might contribute to the increased resistance towards colistin [41]. In contrast, these macromolecules displayed good activity against actively growing, stationary phase as well as the antibiotic-tolerant cells of Gram-negative bacteria at low concentrations. This led us to probe the mechanism of antibacterial activity of this class of cationic amphiphilic molecules against the antibiotic-tolerant cells.

#### Mechanistic investigation against bacterial antibiotic-tolerant cells

**Fig 5:**
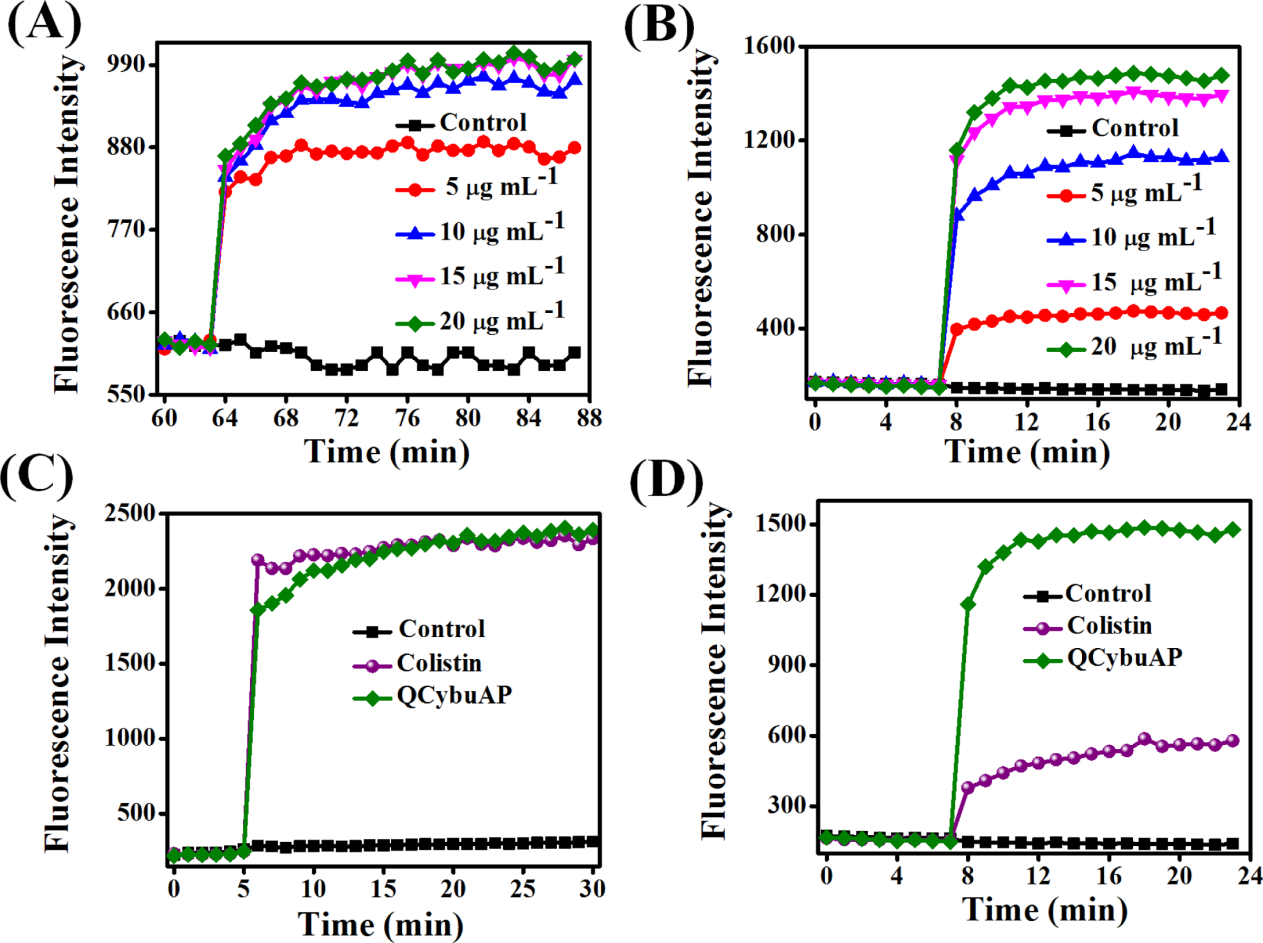
Membrane-active properties of QCybuAP against *E*. *coli*. (A) Concentration dependent effect of QCybuAP on membrane depolarization and (B) membrane permeabilization against antibiotic-tolerant cells; Membrane permeabilization of QCybuAP and colistin against actively growing (C) and antibiotic-tolerant (D**)** cells. The concentrations used were 20 μg mL^-1^.

The membrane-disruptive activity of QCybuAP were studied against bacterial antibiotic-tolerant cells. Membrane depolarization by QCybuAP was studied using a membrane potential sensitive dye, DiSC_3_(5) (3, 3’-dipropylthiadicarbocyanine iodide). QCybuAP showed concentration dependent dissipation of membrane potential against *E*. *coli* (Fig 5A) antibiotic-tolerant cells. Even at 5 μg mL^-1^, the concentration at which complete bacterial killing was observed; QCybuAP dissipated the membrane potential of bacterial antibiotic-tolerant cells.

Membrane permeabilization was studied using propidium iodide (PI). QCybuAP (Fig 5B) and Q*n*-prAP (S2 Fig) permeabilized the cell membrane in concentration dependent manner against *E*. *coli* antibiotic-tolerant cells. QCybuAP even at concentrations less than 5 μg mL^-1^ (S2 Fig) showed membrane-disruptive properties similar to sodium dodecylsulfate (SDS, 20 μg mL^-1^) and 5% ethanol (S2 Fig). This finding that even at concentrations less than 5 μg mL^-1^ (concentration that kills the bacteria) suggests that the membrane-disruptive properties eventually lead to cell death. More importantly, both QCybuAP and colistin at 20 μg mL^-1^ showed equal extent of membrane permeabilization against actively growing cells of *E*. *coli* (Fig 5C). However, against antibiotic-tolerant *E*. *coli*, QCybuAP (20 μg mL^-1^) retained the capability to permeabilize the membranes whereas colistin at the same concentration showed very low extent of membrane permeabilization (Fig 5D). It was reported that colistin when used at high concentration shows membrane permeabilization by increasing PI fluorescence in Gram-negative biofilms where both actively growing and persister cells are present [42].

More importantly, our observations showed that colistin being specific-membrane targeting antibiotic has reduced efficacy against stationary phase and antibiotic-tolerant cells compared to actively growing cells. On the contrary, the cationic amphiphilic macromolecules reported here do not differentiate between the actively growing, stationary phase and antibiotic-tolerant bacteria and employ membrane-active mechanism of bacterial killing. Different strategies have been demonstrated to eradicate antibiotic-tolerant and persister cells in literature [11, 33, 38, 39, 43–47]. These results indicate the potential of membrane-active mode of action for targeting antibiotic-tolerant bacteria.

### Biofilm disruption

#### Gram-negative biofilm disruption in combination with known antibiotics

**Fig 6:**
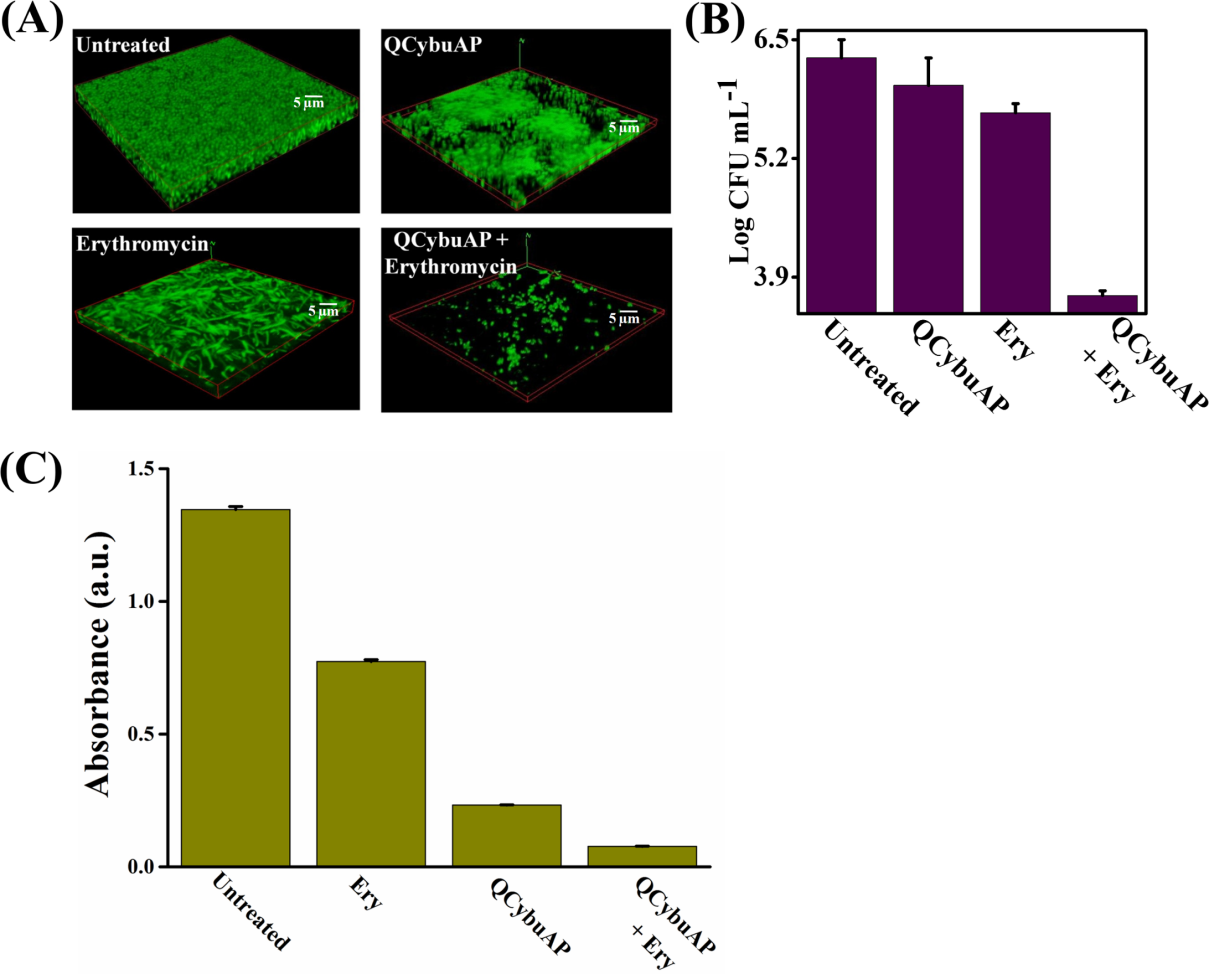
Disruption of *E*. *coli* biofilms. (A) Confocal laser scanning microscopy (CLSM) images of biofilms either treated with QCybuAP (50 μg mL^-1^), erythromycin (Ery, 50 μg mL^-1^) and erythromycin + QCybuAP (50 μg mL^-1^ + 50 μg mL^-1^) or left untreated for 24 h. Biofilms were stained with SYTO 9 dye and each image is a 3D reconstruction of z-stack images; (B) Reduction in bacterial cell counts in biofilms with and without treatment of compounds; (C) Absorbance of the crystal violet (CV) staining of the biofilms.

We studied the effect of restoring the known antibiotics towards tough-to-kill Gram-negative biofilms established on surfaces. Mature *E*. *coli* biofilms were treated with QCybuAP, erythromycin and QCybuAP + erythromycin for 24 h. The confocal laser scanning microscopy (CLSM) images stained with SYTO 9 dye clearly demonstrated the near complete dispersal of biofilm in the presence of QCybuAP + erythromycin (50 μg mL^-1^ + 50 μg mL^-1^) compared to the individual molecules and untreated conditions (Fig 6A). Although QCybuAP displayed activity against stationary phase, antibiotic-tolerant and actively growing bacteria, it was in-active against pre-formed *E*. *coli* biofilms at 50 μg mL^-1^ (Fig 6A). However, QCybuAP + erythromycin (50 μg mL^-1^ + 50 μg mL^-1^) nearly eradicated the pre-formed *E*. *coli* biofilms. On the other hand, treatment with erythromycin (50 μg mL^-1^) alone did not result in biofilm disruption (Fig 6A). Bacterial count enumeration was performed after the treatment of biofilms with the antibacterial agents. QCybuAP + erythromycin (50 μg mL^-1^ + 50 μg mL^-1^) showed nearly 3 log_10_ reduction in bacteria of *E*. *coli* biofilms. In contrast, the individual compounds (QCybuAP and erythromycin) did not show significant reduction in bacterial counts (Fig 6B). Absorbance of the crystal violet staining of biofilms treated with QCybuAP and QCybuAP + erythromycin showed low values compared to erythromycin (Fig 6C). These results showed that the cationic amphiphilic macromolecules restored the efficacy of antibiotic (erythromycin) towards Gram-negative bacterial biofilms.

**Fig 7:**
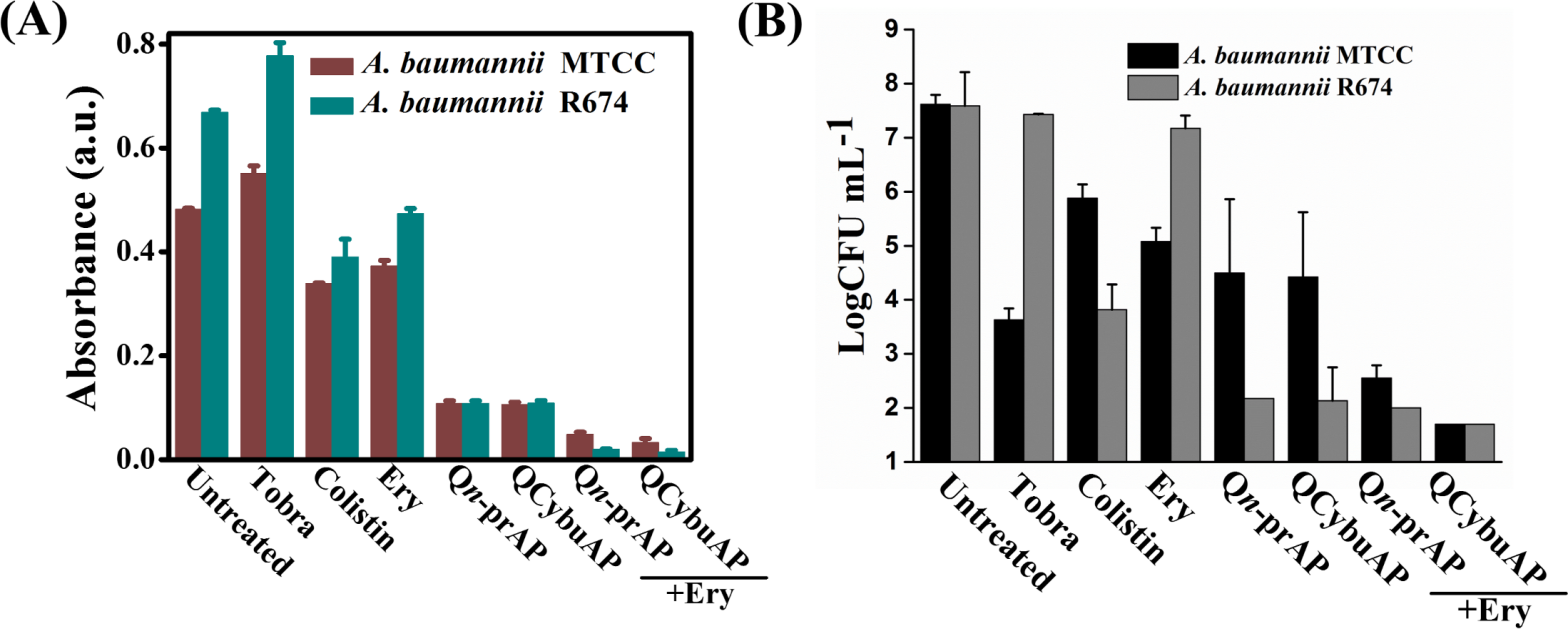
Disruption of *A*. *baumannii* biofilms. (A) Absorbance of crystal violet staining of the *A*. *baumannii* biofilms grown on glass cover slips in presence of colistin (30 μg mL^-1^), Q*n*-prAP and QCybuAP (both at 30 μg mL^-1^), erythromycin (Ery, 30 μg mL^-1^), tobramycin (Tobra, 30 μg mL^-1^), erythromycin + Q*n*-prAP/QCybuAP (30 μg mL^-1^ + 30 μg mL^-1^); (B) Reduction in bacterial cell counts in biofilms with the treatment of colistin (50 μg mL^-1^), Q*n*-prAP and QCybuAP (both at 50 μg mL^-1^), erythromycin (Ery, 50 μg mL^-1^), tobramycin (Tobra, 50 μg mL^-1^), erythromycin + Q*n*-prAP/QCybuAP (50 μg mL^-1^ + 50 μg mL^-1^) compared to untreated control. (limit of detection < 50 CFU/mL).

**Fig 8:**
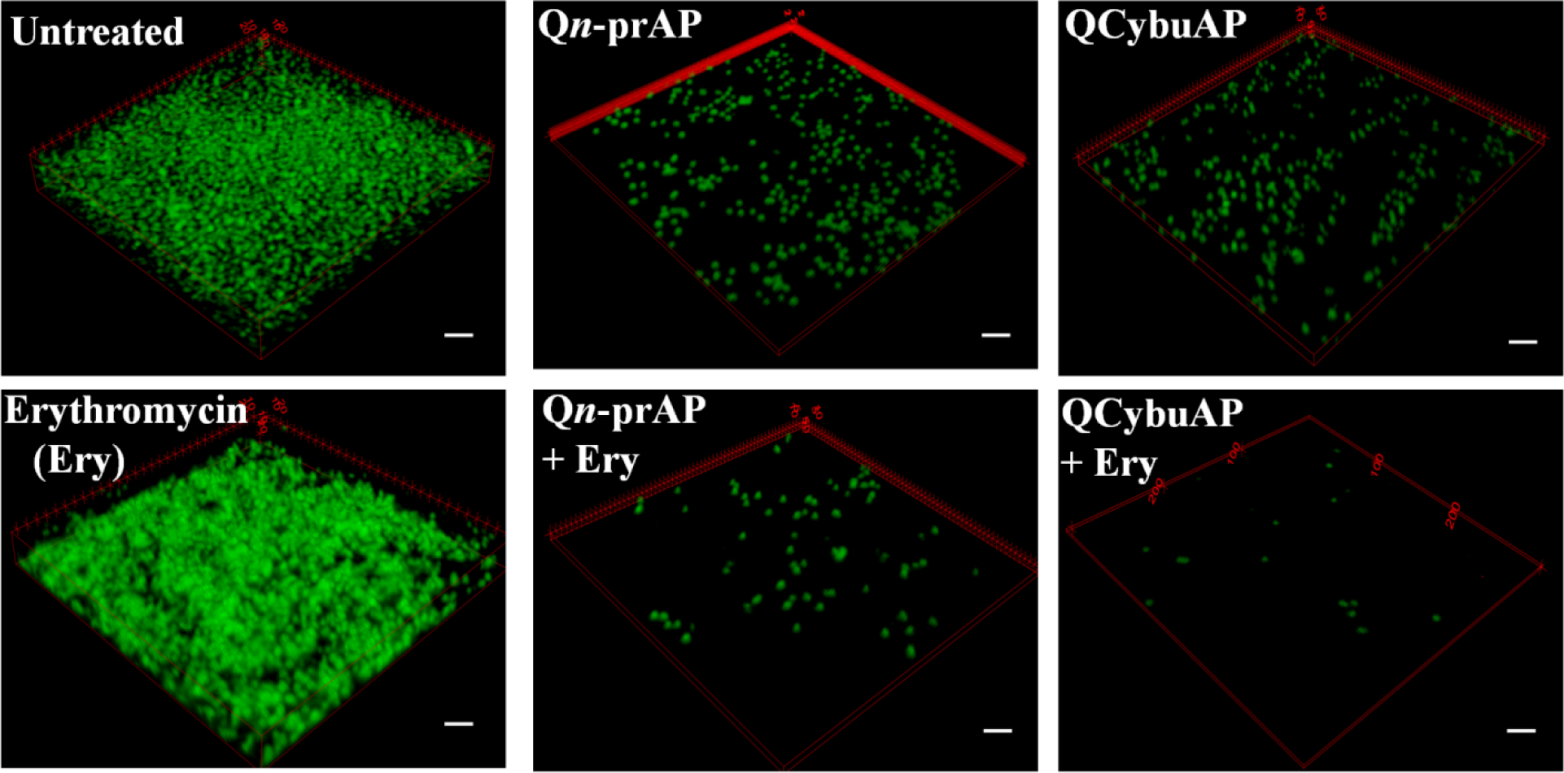
Disruption of *A*. *baumannii* biofilms. Biofilms were treated with colistin (30 μg mL^-1^), Q*n*-prAP and QCybuAP (both at 30 μg mL^-1^), erythromycin (Ery, 30 μg mL^-1^), tobramycin (Tobra, 30 μg mL^-1^), erythromycin + Q*n*-prAP/QCybuAP (30 μg mL^-1^ + 30 μg mL^-1^). Confocal laser scanning microscopy (CLSM) stained with SYTO9 dye. z-stack images were processed using Zeiss LSM software and 3D representation was processed using ImageJ. Scale bars, 5 μm.

Established *A*. *baumannii* MTCC 1425 and *A*. *baumannii* R674 (multi-drug resistant clinical isolate, S1 Table) biofilms were treated with cationic amphiphilic macromolecules (QCybuAP (30 μg mL^-1^), Q*n*-prAP (30 μg mL^-1^)), antibiotics (tobramycin (30 μg mL^-1^), colistin (30 μg mL^-1^), erythromycin (Ery) (30 μg mL^-1^)), in combination (QCybuAP + Ery (30 μg mL^-1^+ 30 μg mL^-1^), Q*n*-prAP + Ery (30 μg mL^-1^+ 30 μg mL^-1^)) for 24 h. Biofilms stained with crystal violet evidently showed that both QCybuAP and Q*n*-prAP alone and in combination with erythromycin showed biofilm disruption properties better than erythromycin, tobramycin and colistin against both *A*. *baumannii* MTCC 1425 and *A*. *baumannii* R674 (Fig 7A & S3 Fig). Bacterial count enumeration of biofilms of both strains were treated with (QCybuAP (50 μg mL^-1^), Q*n*-prAP (50 μg mL^-1^)), antibiotics (tobramycin (50 μg mL^-1^), colistin (50 μg mL^-1^), erythromycin (Ery) (50 μg mL^-1^)), in combination (QCybuAP + Ery (50 μg mL^-1^+ 50 μg mL^-1^), Q*n*-prAP + Ery (50 μg mL^-1^+ 50 μg mL^-1^)) for 24 h. As shown in Fig 7B, the data shows that combination of the agents efficiently kill bacteria compared to the individual polymers and antibiotics. The confocal laser scanning microscopy (CLSM) images of *A*. *baumannii* MTCC 1425 biofilms indicated the dispersal of biofilm in presence of QCybuAP and Q*n*-prAP (both at 30 μg mL^-1^) compared to erythromycin (30 μg mL^-1^) (Fig 8) as described previously [30]. The effect was more pronounced with the combinations, QCybuAP + erythromycin and Q*n*-prAP + erythromycin which showed almost complete eradication of bacteria in the biofilms. The thickness of the biofilms as evident from CLSM images is less for the polymer treated biofilms (2.4 μm) compared to erythromycin treated (7.6 μm) or untreated (18 μm) biofilms as described previously [30]. Polymers in combination with erythromycin showed a thickness of < 2 μm indicating the presence of a monolayer of bacterial cells (Fig 8).

The above results suggest that these macromolecules alone could disrupt *A*. *baumannii* biofilms as also observed earlier [30] and in the present work combination with conventional antibiotics could almost eradicate the biofilms. However, these macromolecules alone could not disrupt the *E*. *coli* biofilms but when combined with erythromycin disrupted the biofilms and killed the biofilm associated bacteria. We believe that these macromolecules might interact with the EPS matrix and weaken the *E*. *coli* biofilms (as shown in Fig 6) and in combination with antibiotic could completely disperse and kill the bacteria. On contrary, the antibiotic alone could neither disrupt the biofilm nor kill the bacteria in it. The observed differences in the potent activity of these macromolecules specifically against *A*. *baumannii* but not *E*. *coli* biofilms might be due to their individual traits of bacteria in the formation of biofilms (may be the EPS differences) as the macromolecules have similar efficacy against the planktonic cells of both the bacteria. We believe that these differences warrant further studies for understanding in detail.

**Fig 9:**
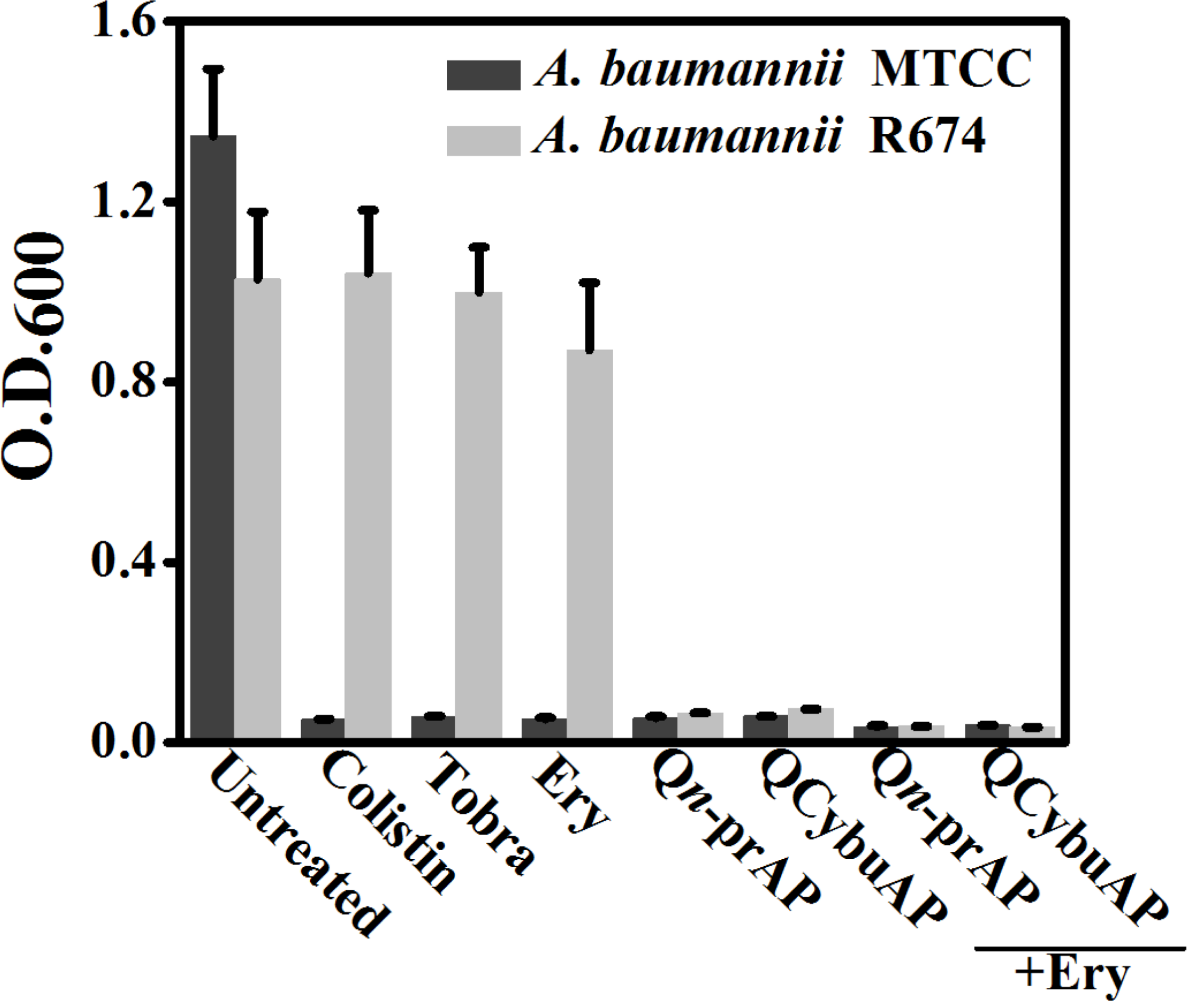
Dispersed cells of *A*. *baumannii* biofilms. O.D._600_ of the planktonic growth of bacteria due to dispersed cells from biofilms in presence of colistin (30 μg mL^-1^), Q*n*-prAP and QCybuAP (both at 30 μg mL^-1^), erythromycin (Ery, 30 μg mL^-1^), tobramycin (Tobra, 30 μg mL^-1^), erythromycin + Q*n*-prAP/QCybuAP (30 μg mL^-1^ + 30 μg mL^-1^).

Dispersed bacteria upon treatment of biofilms with known antibiotics represent a dangerous pool of bacteria that cause more tissue damage and can revert to planktonic condition [48]. We have measured the optical density (O.D._600_) of bacteria that has got dispersed from *A*. *baumannii* biofilms and reverted to planktonic growth stage during the 24 h treatment with antibacterial agents. Interestingly, we found that colistin, tobramycin, erythromycin, QCybuAP and Q*n*-prAP and their combination with existing antibiotics did not allow the growth of planktonic bacteria in the well plates during the 24 h treatment against sensitive strain of *A*. *baumannii* (MTCC 1425) (Fig 9). However, in case of biofilms grown from clinical isolate strain of *A*. *baumannii* R674, known antibiotics did not prevent the growth of planktonic bacteria. Colistin despite being sensitive to *A*. *baumannii* R674 did not arrest the planktonic growth of the dispersed cells from biofilms. Apart from optical density, spot plating of this dispersed bacterial suspension in agar plates showed the growth of bacteria in presence of erythromycin, tobramycin and colistin against both the strains of *A*. *baumannii*. Over all these results suggest that, against the biofilms formed by sensitive strain, all the antibacterial agents prevented the planktonic growth of dispersed bacteria. On contrary, against biofilms formed by resistant strain all the conventional antibiotics including colistin (sensitive to this strain) did not arrest the growth of dispersed cells pointing the phenotypic differences between the actively growing planktonic cells and dispersed cells from biofilms. Cationic amphiphilic macromolecules alone and in combination with known antibiotics not only prevented the planktonic growth (Fig 9) but also eradicated the dispersed cells from biofilms against both sensitive and clinical isolate *A*. *baumannii* strains. These results led to the understanding, that unlike known antibiotics, the membrane-active molecules in combination with antibiotics not only disrupted the biofilms but also arrested the planktonic growth of dispersed bacteria from biofilms. Hence, the combination approach is useful for reducing the spread of highly virulent dispersed cells.

#### Membrane-active macromolecules potentiate efflux antibiotics to Gram-negative bacteria

Most of the Gram-negative bacteria are inherently resistant to antibiotics such as erythromycin and rifampicin (both are active against Gram-positive bacteria) and these bacteria acquire resistance to tetracyclines by excluding them through efflux pumps (mostly RND (resistance nodulation division) family) [4, 49]. The proton motive force, an electrochemical gradient in which the movement of hydrogen ions drives transport of the substrate, drives efflux through RND family pumps [49]. Since the activity of the efflux pumps depend on the proton motive force across the bacterial cell membrane, we envisaged that these cationic amphiphilic macromolecules which can dissipate the membrane potential (Fig 5) might be able to restore the efficacy of these conventional antibiotics. *E*. *coli* and *A*. *baumannii* were found to be inherently resistant to erythromycin (MIC = 38 μg mL^-1^ and 6.2 μg mL^-1^) and rifampicin (MIC = 8 μg mL^-1^ and 3.1 μg mL^-1^) (S2 Table). We performed chequer-board assays to determine the synergy profiles between these known antibiotics and molecules (QCybuAP and Q*n*-prAP) against both the bacteria in planktonic state (S4, S5, S6, S7, S8, S9 & S10 Figs). We found that the macromolecules restored the efficacy of existing antibiotics to as low as 0.1 μg mL^-1^ (S2 and S3 Tables). Using the fractional inhibitory concentration (FIC = [X]/MIC_X_, where [X] is the lowest inhibitory concentration of compound 1 in the presence of the compound 2), a combination was called synergistic when the FIC index (FICI = FIC_compound1_ + FIC_compound2_) was ≤ 0.5 [34]. Both the molecules showed synergistic profiles with erythromycin and rifampicin against *E*. *coli* and *A*. *baumannii* (FICI in the range of 0.1–0.5) (S2 Table). Unlike erythromycin and rifampicin that are inherently resistant, Gram-negative bacteria acquire resistance to tetracycline antibiotics and we have recently reported that these cationic macromolecules resensitized planktonic cells of NDM-1 producing Gram-negative clinical isolates to tetracycline antibiotics [34]. These results suggested that this class of macromolecules have the potential to restore the efficacy of established antibiotics.

#### Plausible mechanism of potentiating the conventional antibiotics

In a pursuit to determine the role of efflux pumps in the mechanism of antibiotic resensitization, we investigated the antibiotic restoration ability of cationic macromolecules and PAβN (phenyl arginine-β-naphthylamide) for linezolid and vancomycin. PAβN, a very well-known efflux pump inhibitor of Gram-negative bacteria has been shown to restore the efficacy of known antibiotics [50]. Linezolid, an oxazolidinone class of antibiotic, is ineffective against Gram-negative bacteria due to resistance by efflux pumps and other mechanisms [4]. Gram-negative bacteria are also resistant to vancomycin, a glycopeptide antibiotic, due to its inability to cross the additional outer membrane [4]. We have observed that *E*. *coli* was inherently resistant to linezolid and vancomycin with MIC of 100 μg mL^-1^ and >100 μg mL^-1^ respectively (S2 and S4 Tables). In presence of both Q*n*-prAP and PAβN, MIC of linezolid was decreased to 12.5–25 μg mL^-1^ whereas no effect was observed for vancomycin against *E*. *coli* (S11, S12, S13 & S14 Figs). Moreover, the MIC of erythromycin and rifampicin were decreased to 0.1–0.8 μg mL^-1^ in presence of PAβN towards *E*. *coli* (S4 Table and S11, S12, S13 & S14 Figs). We have also observed that membrane-active macromolecules and PAβN increased the uptake of tetracycline antibiotics (efflux pumps are the mechanism of resistance) in planktonic cells of Gram-negative bacteria as the mechanism of resensitization (S15 Fig). These results indicated two observations: i) these macromolecules and PAβN restore the efficacy of known antibiotics that are excluded by efflux pumps of Gram-negative bacteria and ii) both these agents do not restore the efficacy of antibiotics for e.g. linezolid and vancomycin that possess mechanisms of resistance other than efflux pumps. These macromolecules dissipating the transmembrane potential thereby affect the efflux pump activity and also cause membrane permeabilization. The present results make us believe that these macromolecules indirectly inhibit the action of efflux pumps (unlike PAβN, a classical efflux pump inhibitor) and restore the efficacy of efflux antibiotics from Gram-negative bacteria [51] but this certainly needs further investigations. Vaara et al. also have shown that modified polymyxin derivatives show strong synergism with rifampicin and clarithromycin against Gram-negative bacteria [52]. Wright and co-workers have elegantly shown that a class of anthracycline derivatives dissipate the membrane potential and inhibit the efflux pump activity of Gram-negative bacteria [53]. These derivatives were shown to potentiate the efflux antibiotics to Gram-negative bacteria [53]. Similar observations were made by Mor and co-workers [54] and others [55]. Taken together, these results suggest that membrane-active molecules can restore the efficacy of conventional antibiotics thereby providing a backdoor entry to antibiotics that are otherwise excluded from efflux pumps of Gram-negative bacteria.

The highlight of the results is that planktonic Gram-negative bacteria is inherently resistant to erythromycin and rifampicin due to their exclusion by efflux pumps and the macromolecules showed efficacy in combination with these known antibiotics against tough-to-kill biofilms. These results also suggest that known antibiotics are less effective against the metabolically inactive antibiotic-tolerant cells as well as biofilms. In contrast, the cationic amphiphilic macromolecules displayed good activity against actively growing, stationary phase as well as the antibiotic-tolerant cells of Gram-negative bacteria at low concentrations with membrane-active mechanism of action. In the context of increasing evidence for role of efflux pumps in the tolerance of biofilms to conventional antibiotics, the use of efflux pump inhibitors (such as PAβN) alone or in combination with known antibiotics has been shown to disrupt biofilms [49, 50, 56, 57]. Hence, we believe that, as shown above, the indirect inhibition of efflux pumps due to dissipation of membrane potential by these macromolecules might have improved the antibiotic efficacy even in biofilms leading to disruption and killing of bacteria. The cationic charge of these molecules might interact with the negatively charged EPS of biofilms thereby weakening the biofilms followed by killing the bacteria in combination with known antibiotics. Molecules alone or in combination with known antibiotics with activity against antibiotic-tolerant cells and biofilms have extensively been reported [38, 39, 42, 58–67]. The present findings highlight the importance of non-specific targeting of membrane for the elimination of bacterial antibiotic-tolerant cells and eradication of biofilms.

#### Drug resistance studies

**Fig 10:**
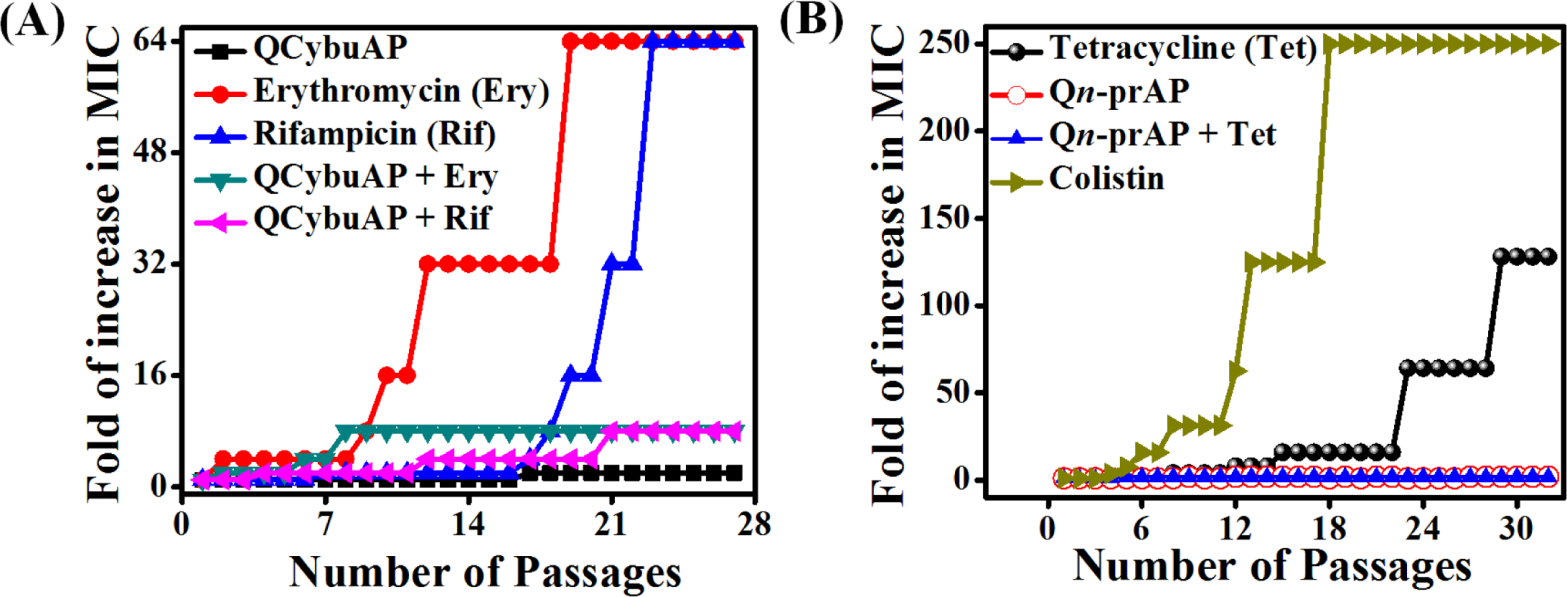
Development of bacterial resistance. (A) *A*. *baumannii* did not develop resistance to QCybuAP but a very high and rapid resistance was observed for known antibiotics with an increase in MIC up to 64 fold after 28 passages. Macromolecules delay the development of resistance to both the known antibiotics with only 8-fold increase in MIC even after 28 passages. (B) *E*. *coli* developed rapid resistance to colistin and tetracycline but not to Q*n*-prAP and Q*n*-prAP + tetracycline.

*A*. *baumannii* was found to be inherently resistant to erythromycin and rifampicin. We have evaluated the ability to develop bacterial resistance to erythromycin and rifampicin in presence of QCybuAP against *A*. *baumannii* (Fig 10A). Erythromycin and rifampicin showed 64-fold increase in MIC and on the other hand no increase in MIC was observed for QCybuAP against *A*. *baumannii* even after 28 passages (Fig 10A). Interestingly, in presence of the macromolecule, *A*. *baumannii* had low tendency for antibiotic resistance with only 8-fold increase in the MIC of known antibiotics at the end of 28 passages. We have also observed that this macromolecule stopped the development of bacterial resistance to tetracycline in *E*. *coli* (Fig 10B). Bacteria developed rapid resistance to colistin with increase in MIC up to 250 fold against *E*. *coli* (Fig 10B) and it has been known that *A*. *baumannii* also developed rapid resistance to colistin [68]. Overall, these results suggested that macromolecules prevented the development of bacterial resistance for tetracycline antibiotics and delayed for erythromycin and rifampicin in Gram-negative bacteria.

### Animal studies

#### *In-vivo* toxicity

We performed in-vivo toxicity studies of QCybuAP and Q*n*-prAP in mice models. Administration of Q*n*-prAP or QCybuAP at 55 mg kg^-1^ through subcutaneous (s.c.) route showed no lethality. Experiments performed to assess the *in-vivo* toxicity of Q*n*-prAP after single-dose intravenous (i.v.) administration to mice resulted in LD_50_ values of 20 mg kg^-1^. This compares favorably with clinically approved antibiotics such as polymixins, which work by a comparable cell-lytic mechanism as AMPs, and have lower LD_50_ levels of approximately 8–10 mg kg^-1^ with reported neuro- and nephro-toxicity. We have also tested the toxicity of the Q*n*-prAP through single dose intraperitoneal (i.p.) injection into the mice and found no lethal effects up to 17.5 mg kg^-1^. For the combination efficacy of antibiotics and polymers, the mice were given a single i.v. injection of combination of doxycycline and Q*n*-prAP. It was observed that the mice showed no lethal effects after injection of doxycycline + Q*n*-prAP up to 100 mg kg^-1^ + 15 mg kg^-1^ and also doxycycline (100 mg kg^-1^) alone. This showed that these polymers alone and in combination with antibiotics have low toxicity in mice models and have a good safety profile required for therapeutic applications *in*-*vivo*. Macromolecules can act as non-absorbable drugs for safe topical treatment of bacterial infections, [69, 70] such as burn/surgical wound infections as they offer advantages like minimum systemic toxicity.

#### Acute burn wound infection

**Fig 11:**
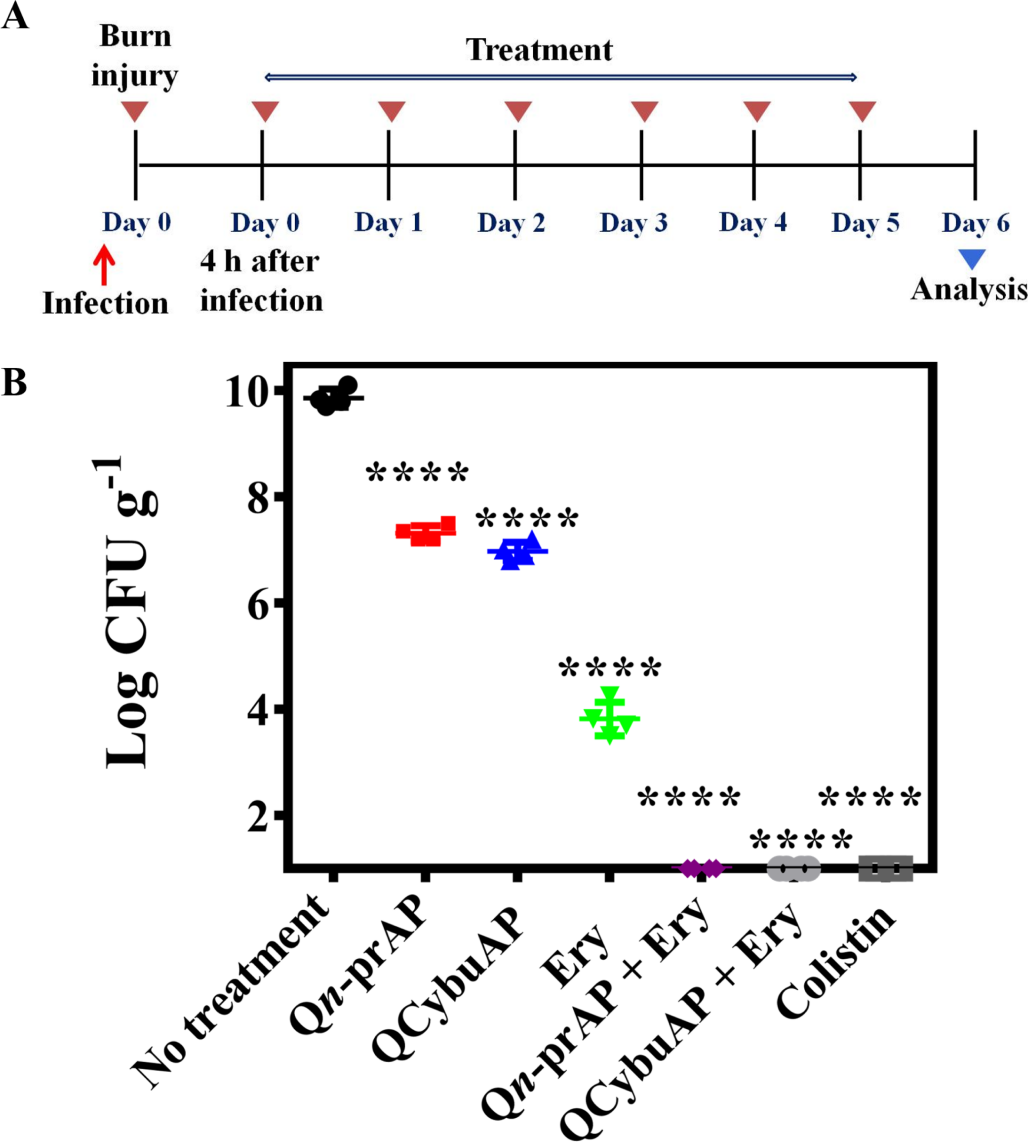
*In-vivo A*. *baumannii* burn wound infection. (A). Experimental plan. (B). Mice (n = 4) were treated with erythromycin (Ery, 20 mg kg^-1^), QCybuAP (50 mg kg ^-1^), Q*n*-prAP (50 mg kg^-1^), Q*n*-prAP + Ery (50 mg kg^-1^ + 20 mg kg^-1^), QCybuAP + Ery (50 mg kg^-1^ + 20 mg kg^-1^) and colistin (5 mg kg^-1^). Combination treatment and colistin showed decrease below detection limits (< 50 CFU mL^-1^). P value was calculated using one way ANOVA (Dunnett's Multiple Comparison Test) between the control group and treatment groups and a value of P < 0.05 was considered significant.

**Fig 12:**
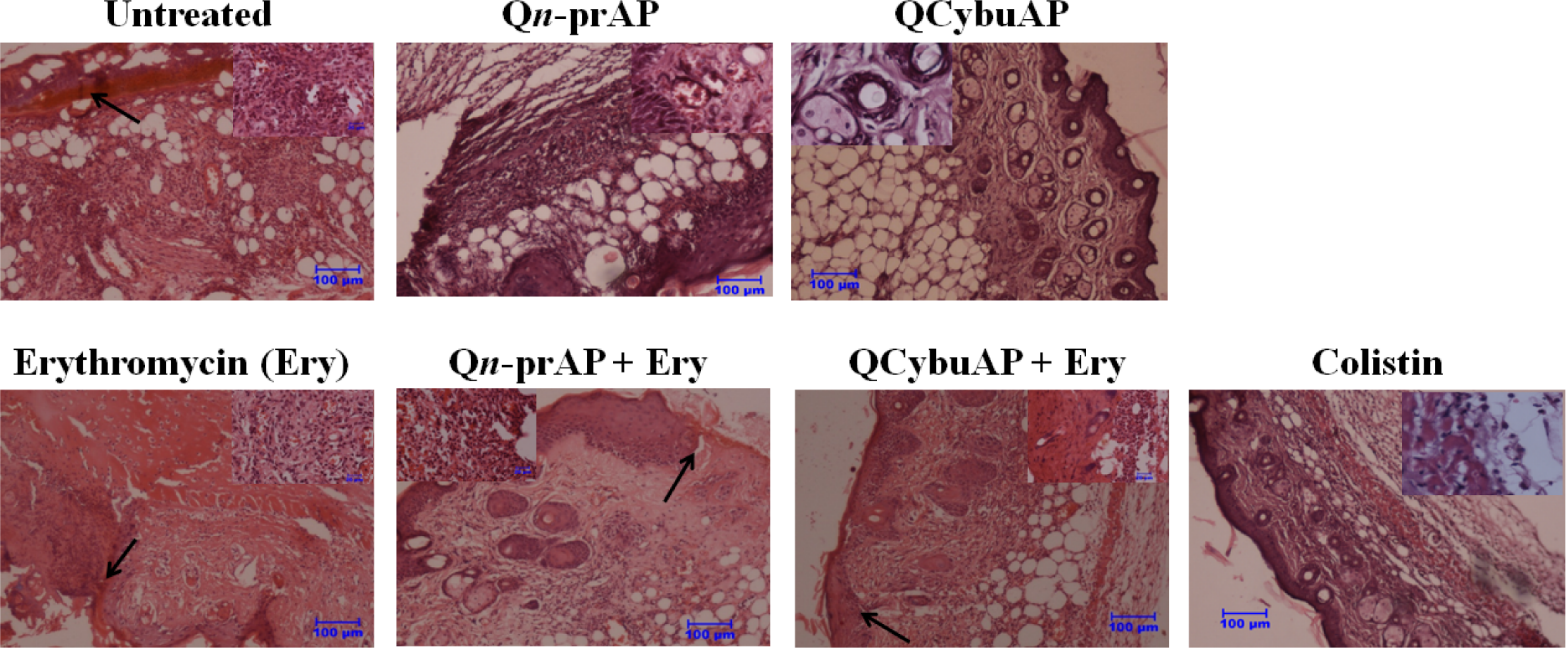
Histopathology analysis. Scale bar = 100 μm (inset, 20 μm).

The *in-vivo* antibacterial efficacy studies were performed in mice using burn wound infection model [36] against *A*. *baumannii* [71]. Burn wounds were created on the back of the mice using heated brass bars and infected the burn wounds with *A*. *baumannii* (Fig 11A). Treatment was given every 24 h for 5 days. Mice treated with Q*n*-prAP, QCybuAP (both at 50 mg kg^-1^) and erythromycin (20 mg kg^-1^) showed 3 log_10_, 3 log_10_ and 6 log_10_ reductions (p ≤ 0.0001) respectively in bacterial burden compared to the untreated mice (10 log_10_) (Fig 11B). Q*n*-prAP + erythromycin (50 mg kg^-1^ + 20 mg kg^-1^), QCybuAP + erythromycin (50 mg kg^-1^ + 20 mg kg^-1^) and colistin (5 mg kg^-1^) had decreased the bacterial burden down to the detection limits (< 50 CFU mL^-1^) (Fig 11B). Histopathology analysis of untreated control showed burn wound area with numerous bacterial cells with proteinaceous exudates (arrow) along with severe infiltration of inflammatory cells mainly neutrophils which are degenerating (inset) (Fig 12). There was loss of squamous epithelial cells, sweat gland, sebaceous gland and hair follicles along with architecture of skin tissue. Erythromycin treated group showed the process of healing which is evident by presence of fibrous tissue and squamous epithelial cells from the surrounding area (arrow) with severe infiltration of inflammatory cells mainly neutrophils and also congestion of blood vessels (inset). Q*n*-prAP treated group showed moderate infiltration of inflammatory cells with congestion of blood vessels (inset) and regeneration of stratified squamous epithelial cells with loss of sebaceous and sweat glands. Mice that received Q*n*-prAP +Erythromycin treatment showed regeneration of squamous epithelial cell layer over the burn wound area along with fibrous tissue (arrow), appearance of sweat and sebaceous glands, and hair follilces with moderate infiltration of neutrophils in the subepithelial layer (inset). QCybuAP treated mice indicated regeneration of the skin with squamous epithelial cells with keratin layers, sebaceous gland, sweat gland and hair follicles (inset). Mice that received QCybuAP +Erythromycin treatment displayed regeneration of stratified squamous epithelial cells over the burn wound area (arrow) with appearance of hair follicles, sebaceous and sweat glands, along with infiltration of neutrophils beneath the fibrous tissue (inset). Colistin treated skin showed moderate regeneration of squamous epithelial cells with infiltration of inflammatory cells and red blood cells (inset) (Fig 12).

**Fig 13:**
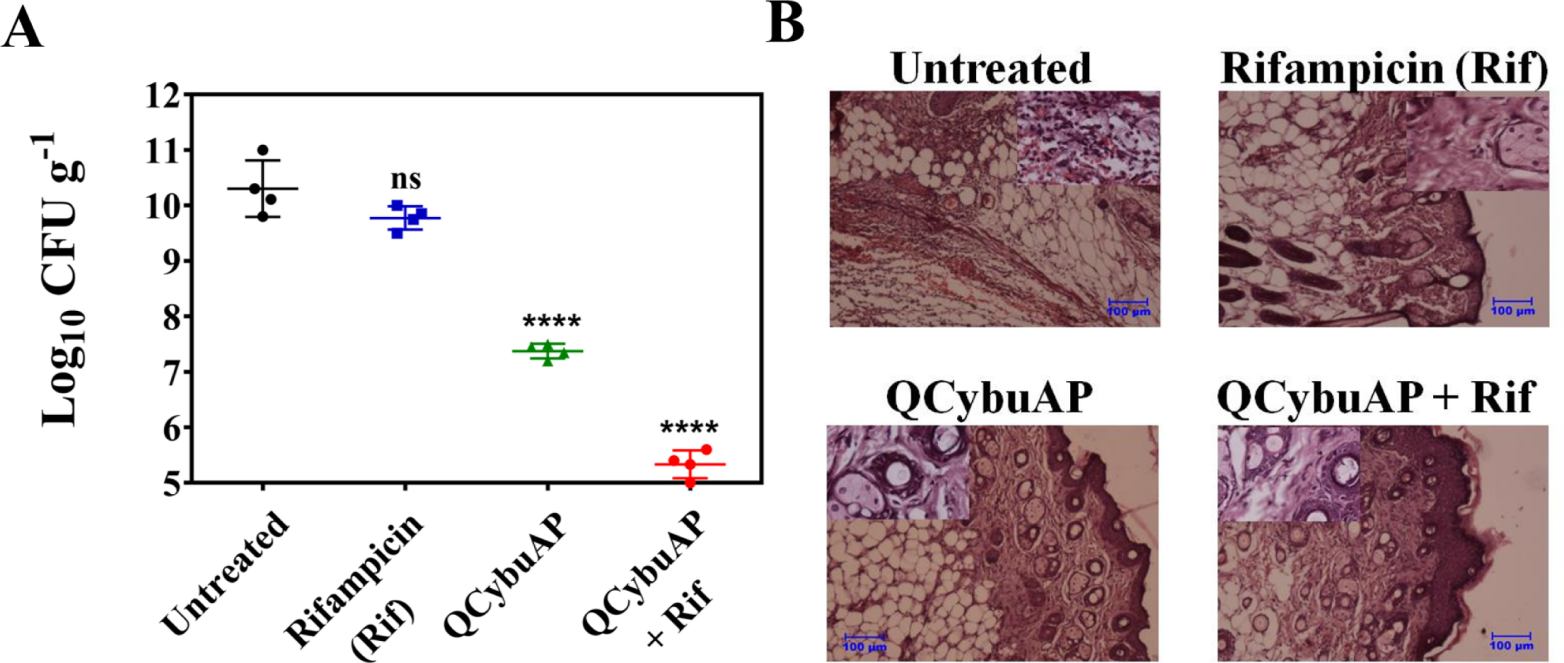
*In-vivo A*. *baumannii* burn wound infection. (A**)** Mice (n = 4) were treated with rifampicin (rif, 5 mg kg^-1^), QCybuAP (50 mg kg^-1^) and QCybuAP + rif (50 mg kg^-1^ + 5 mg kg^-1^). P value was calculated using one way ANOVA (Dunnett's Multiple Comparison Test) between the control group and treatment groups and a value of P < 0.05 was considered significant. (B) Histopathology analysis. Scale bar = 100 μm (inset 20 μm).

Similarly, as above, in another acute burn wound *A*. *baumannii* infection, mice were treated with QCybuAP (50 mg kg^-1^), rifampicin (5 mg kg^-1^) and QCybuAP + rifampicin (50 mg kg^-1^+ 5 mg kg^-1^) every 24 h for 5 days (Fig 13A). Six days post-infection, untreated mice and even rifampicin had a very high bacterial burden of 10–11 log(CFU/g). On the other hand, mice treated with QCybuAP showed (p ≤ 0.0001) decrease of ~3 log(CFU/g) in bacterial burden compared to the untreated mice. More importantly, mice receiving QCybuAP + rifampicin had a very high significant reduction (p ≤ 0.0001) of 6 log(CFU/g) in the bacterial burden compared to the untreated conditions (Fig 13A). Skin histolopathology of control mice without treatment showed infiltration of inflammatory cells mostly neutrophils and mononuclear cells (inset) and damage to squamous stratified epithelial cells (Fig 13B). Rifampicin treated showing moderate regeneration of stratified squamous epithelial cells with sweat gland and sebaceous glands (inset). QCybuAP treated mice showing regeneration of the skin with squamous epithelial cells with keratin layers, sebaceous gland, sweat gland and hair follicles (inset). QCybuAP + rifampicin treated skin showed recovery from the burn wound with numerous keratin layers, sweat and sebaceous glands and hair follicles (inset) (Fig 13B).

#### Murine surgical chronic wound infection

**Fig 14:**
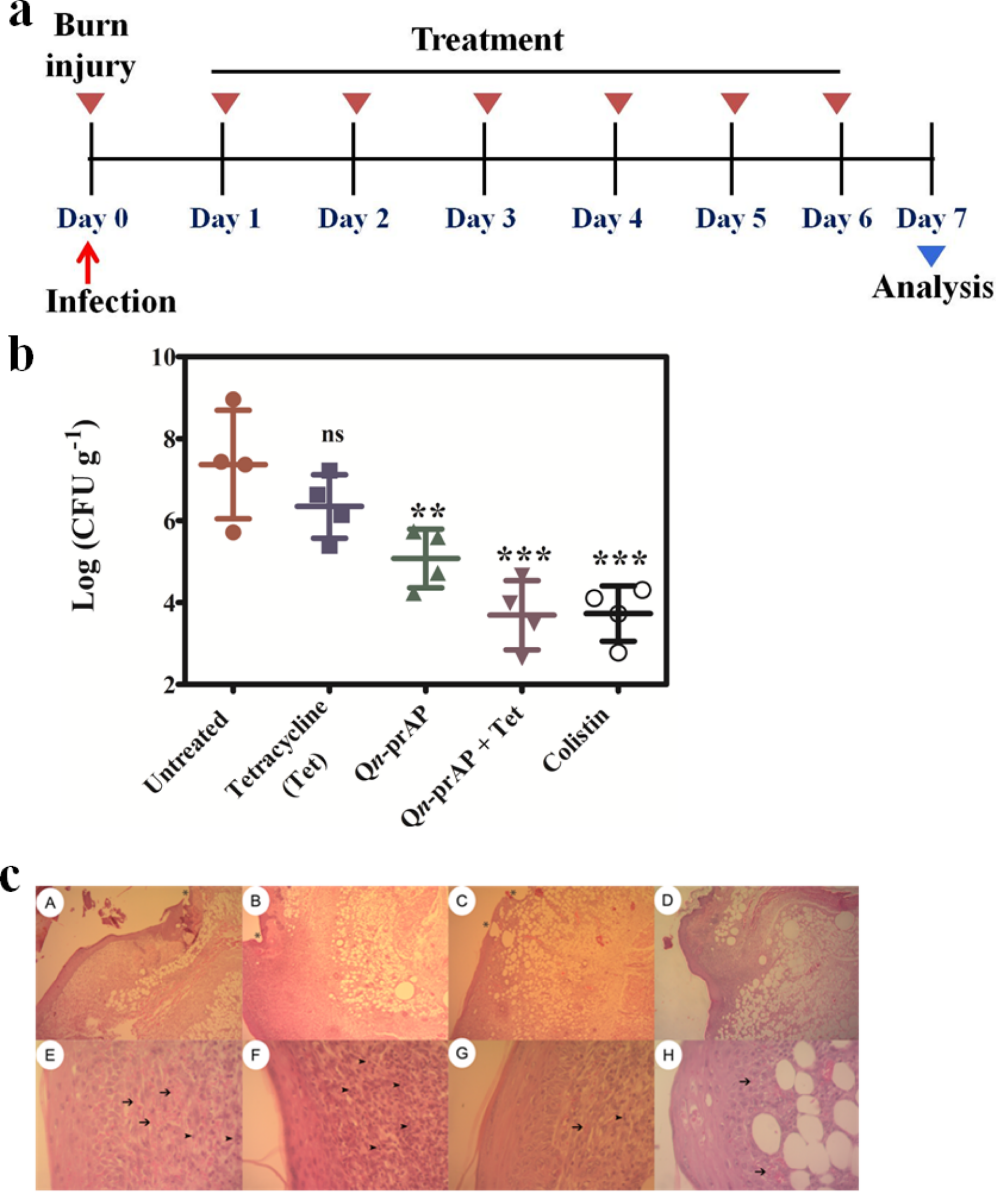
*In-vivo* anti-infective activity against *K*. *pneumoniae* (carbapenem and tetracycline resistant clinical isolate, KPC) surgical wound infection. **a.** Experimental model. **b.** Mice (n = 4) were treated with colistin (5 mg kg^-1^), tetracycline (Tet) (100 mg kg^-1^), QCybuAP (50 mg kg^-1^) and QCybuAP + Tet (50 mg kg^-1^+ 100 mg kg^-1^). P value was calculated using one way ANOVA (Dunnett's Multiple Comparison Test) between the control group and treatment groups and a value of P < 0.05 was considered significant. **c**. Histopathology analyses of mice wounds untreated (A and E) and treated with colistin (B and F), Q*n*-prAP (C and G) and Q*n*-prAP + Tetracycline (D and H).

We used a carbapenemase producing strain of *K*. *pneumoniae* (KPC) in a murine surgical wound infection model [37] for testing the efficacy of Q*n*- prAP in combination with antibiotics. The KPC gene screening by PCR amplification [72] and the resulting gel electrophoresis data along with the strain susceptibility data are provided in the supporting information (S16 Fig & S5 Table). Surgical wounds were created on the dorsal surface of the mice (n = 4) with 6 mm biopsy punch needles and infected with carbapenem and tetracycline resistant *K*. *pneumoniae* (KPC). Wounds were left untreated for 24 h to simulate the conditions for formation of biofilms [30, 73] (Fig 14A**).** Treatment started 24 h post-infection and thereafter every 24 h for 7 days with colistin (5 mg kg^-1^), tetracycline (100 mg kg^-1^), Q*n*-prAP (50 mg kg^-1^) and Q*n*-prAP + tetracycline (50 mg kg^-1^ + 100 mg kg^-1^). Mice that were left untreated had a very high bacterial burden of 8–9 log(CFU/g). Tetracycline treatment was found have no significant effect on the wounds whereas treatment with Q*n*-prAP significantly (p ≤ 0.01) reduced the bacterial burden by 3 log(CFU/g) after 7 days. Interestingly, tetracycline in combination with Q*n*-prAP drastically reduced (p ≤ 0.001) the bacterial burden by 5–6 log(CFU/g) similar to colistin (Fig 14B).

Histopathology results of the skin infected mice showed clear damage to tissue including little epithelialization, focal inflammation with intense polymorphonuclear (PMNs) and mononuclear cell infiltration, loose irregular connective tissue and initial neovascularisation (Fig 14C). Skin histolopathology of mice treated with colistin, Q*n*-prAP and Q*n*-prAP + tetracycline showed a healing process at infection site damaged by the pathogen (Fig 14C). These results indicated that this combination is equally effective as colistin towards topical treatment of Gram-negative bacterial infections. However, the development of bacterial resistance to colistin is a major drawback for the treatment. On the contrary, lack of resistance development for our combination approach highlights its use for the treatment. These results suggest that this combination of membrane-active polymers in combination with antibiotics has enormous potential for the treatment of Gram-negative infections. However, these preliminary experiments were carried out with n = 4 mice and detailed *in-vivo* experiments will be required.

## Conclusions

In conclusion, we have demonstrated excellent antibacterial activity of cationic amphiphilic macromolecules against stationary phase and antibiotic-tolerant bacteria surpassing the conventional antibiotics, kanamycin and colistin. These macromolecules potentiated the antibiotics to disrupt and kill Gram-negative biofilms. The combination of macromolecules and antibiotics also arrested the planktonic growth of dispersed cells from biofilms unlike colistin and tobramycin. The non-specific targeting of bacterial cell membrane by the macromolecule is the key factor in driving their activity towards bacterial antibiotic-tolerant cells and their antibiotic combination efficacy against Gram-negative bacteria. More importantly, the macromolecules in combination with antibiotics reduced the bacterial burden in burn and surgical wound infections caused by Gram-negative bacteria. Furthermore, the macromolecules delayed the bacterial resistance to known antibiotics. Collectively, these findings support the potential implications of this combination approach of membrane-active macromolecule and antibiotics for the topical treatment of Gram-negative bacterial infections.

## Supporting information

S1 Checklist (DOC)

S1 Fig Time-kill curves for the generation of *E*. *coli* antibiotic-tolerant cells. Stationary phase cells were treated with ampicillin to obtain surviving antibiotic-tolerant cells.
(TIF)

S2 Fig Membrane permeabilization of *E*. *coli* antibiotic-tolerant cells. Concentration dependent effect of QCybuAP (A) and (B) Q*n*-prAP and also positive controls (C).
(TIF)

S3 Fig Disruption of *A*. *baumannii* biofilms. Biofilms grown on glass cover slips were treated in presence of colistin (30 μg mL^-1^), Q*n*-prAP and QCybuAP (both at 30 μg mL^-1^), erythromycin (Ery, 30 μg mL^-1^), tobramycin (Tobra, 30 μg mL^-1^), erythromycin + Q*n*-prAP/QCybuAP (30 μg mL^-1^ + 30 μg mL^-1^) or left untreated for 24 h. Crystal violet staining of the glass cover slips was performed and the dye was dissolved in 95% ethanol. The solution was transferred to a fresh 96-well plate and the image of the plate was taken with a digital camera.
(TIF)

S4 Fig Chequer board assays. Antibacterial activity of erythromycin and Q*n*-prAP in combination with erythromycin against *E*. *coli*.
(TIF)

S5 Fig Chequer board assays. Antibacterial activity of Rifampicin and Q*n*-prAP in combination with rifampicin against *E*. *coli*.
(TIF)

S6 Fig Chequer board assays. Antibacterial activity of Q*n*-prAP and QCybyAP against *E*. *coli*.
(TIF)

S7 Fig Chequer board assays. Antibacterial activity of QCybuAP in combination with erythromycin and rifampicin against *E*. *coli*.
(TIF)

S8 Fig Chequer board assays. Antibacterial activity of QCybuAP and Q*n*-prAP in combination with erythromycin against A. *baumannii*.
(TIF)

S9 Fig Chequer board assays. Antibacterial activity of QCybuAP and Q*n*-prAP in combination with rifampicin against A. *baumannii*.
(TIF)

S10 Fig Chequer board assays. Antibacterial activity of QCybuAP, Q*n*-prAP, rifampicin and erythromycin against A. *baumannii*.
(TIF)

S11 Fig Chequer board assays. Antibacterial activity of in linezolid and Q*n*-prAP combination with Linezolid against *E*. *coli*.
(TIF)

S12 Fig Chequer board assays. Antibacterial activity of in vancomycin and Q*n*-prAP combination with vancomycin against *E*. *coli*.
(TIF)

S13 Fig Chequer board assays. Antibacterial activity of PAβN and PAβN in combination with erythromycin against *E*. *coli*.
(TIF)

S14 Fig Chequer board assays. Antibacterial activity of PAβN in combination with rifampicin, linezolid and vancomycin against *E*. *coli*.
(TIF)

S15 Fig Uptake of tetracycline in *bla*_NDM-1_ *E*. *coli* (R3336). Uptake of tetracycline by increase in its fluorescence in presence of Q*n*-prAP (20 μg mL^-1^) and PAβN (50 μg mL^-1^). Tetracycline was used at 100 μg mL^-1^. Relative fluorescence was calculated by subtracting the fluorescence without the bacteria from the fluorescence of bacteria containing samples.
(TIF)

S16 Fig Electrophoresis results for the *bla*KPC gene screening by PCR amplification (DNA fragment at 1011bp). M-100 bp DNA ladder, **Lane 1**, positive control—*K*. *pneumoniae* IOC4955; **Lane 2**, negative control—*K*. *pneumoniae* ATCC700603; **Lane 3**, Negative clinical isolate—1; **Lane 4**, 003259271—*K*. *pneumoniae*; **Lane 5**, Negative clinical isolate– 2; **Lane 6**, Positive clinical isolate—1; **Lane 7**, Negative clinical isolate– 3; **Lane 8**, Positive clinical isolate—2.
(TIF)

S1 Table Antibiotic susceptibility data of *A*. *baumannii* R674 clinical isolate. (DOCX)

S2 Table Antibacterial efficacy of polymers in combination with antibiotics. (DOCX)

S3 Table Antibacterial efficacy in nutrient broth. (DOCX)

S4 Table Role of efflux pumps in antibiotic resensitization towards Gram-negative bacteria. (DOCX)

S5 Table Antibiotic susceptibility data of *K*. *pneumoniae-*003259271 (carbapenemase producing strain, KPC) clinical isolate. (DOCX)
