# Supplementary material for: Membrane-active macromolecules kill antibiotic-tolerant bacteria and potentiate antibiotics towards Gram-negative bacteria
Source: PLoS One. 2017 Aug 24;12(8):e0183263. doi: 10.1371/journal.pone.0183263 (PMC5570306; doi:10.1371/journal.pone.0183263)
Supplement: S1 Checklist — (DOC) [file pone.0183263.s001.doc]

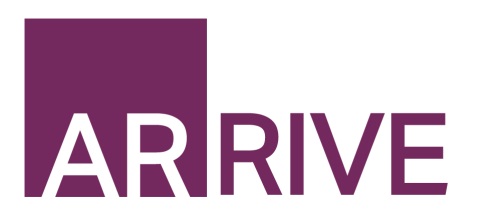


The ARRIVE Guidelines Checklist

Animal Research: Reporting In Vivo Experiments

Carol Kilkenny^1^, William J Browne^2^, Innes C Cuthill^3^, Michael Emerson^4^ and Douglas G Altman^5^

*^1^The National Centre for the Replacement, Refinement and Reduction of Animals in Research, London, UK, ^2^School of Veterinary Science, University of Bristol, Bristol, UK, ^3^School of Biological Sciences, University of Bristol, Bristol, UK, ^4^National Heart and Lung Institute, Imperial College London, UK, ^5^Centre for Statistics in Medicine, University of Oxford, Oxford, UK.*

|  | ITEM | RECOMMENDATION | Section/ Paragraph |
| --- | --- | --- | --- |
| 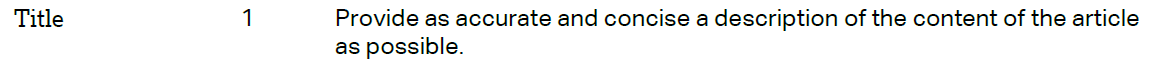 | | | See the attachment |
| 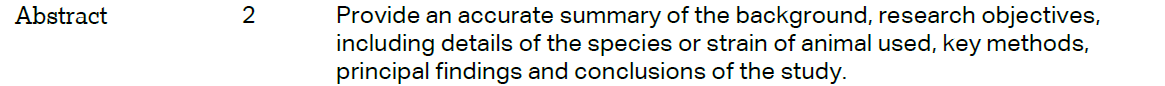 | | | See the attachment |
| INTRODUCTION | | |  |
| 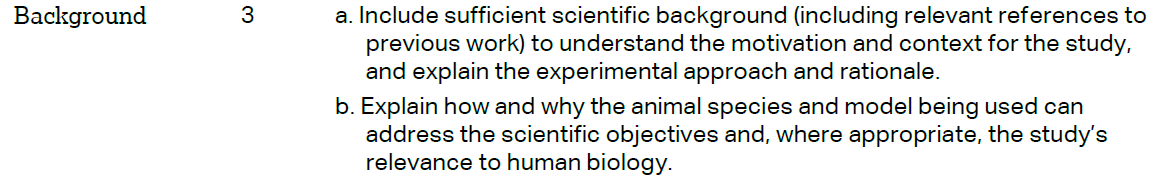 | | | See the attachment |
| 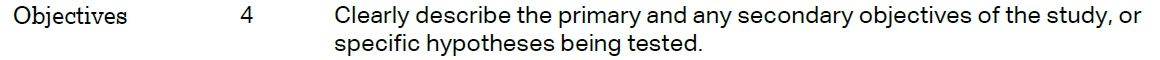 | | | See the attachment |
| METHODS | | |  |
| 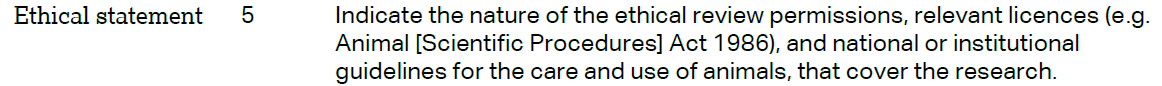 | | | Materials and Methods |
| 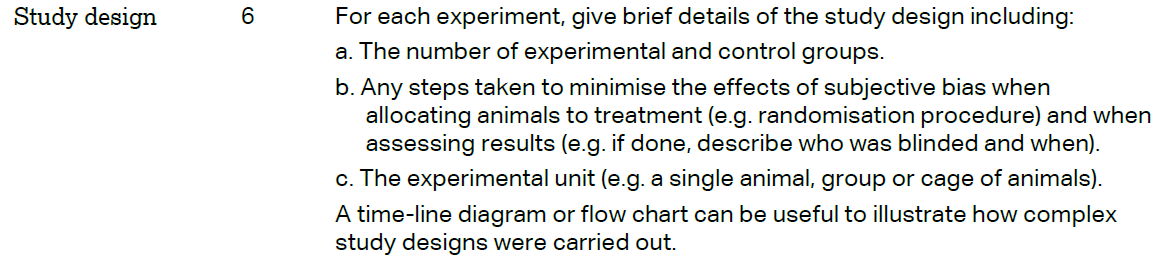 | | | Materials and Methods |
| 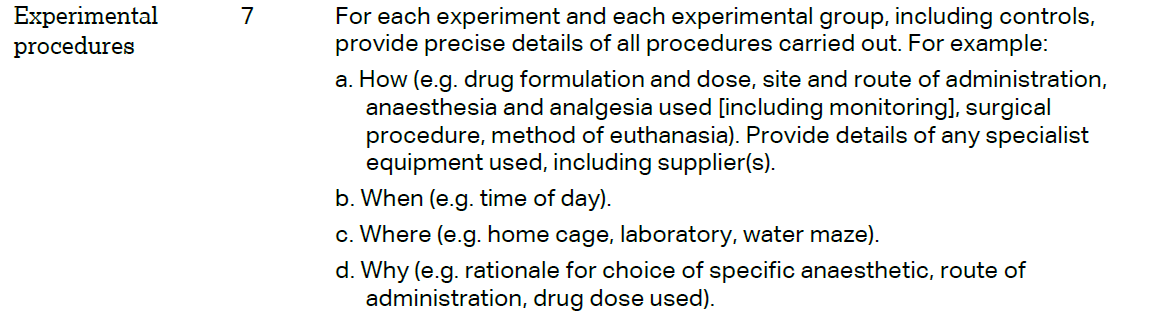 | | | Materials and Methods  All the procedures comply with the OECD guidelines (OECD 425) |
| 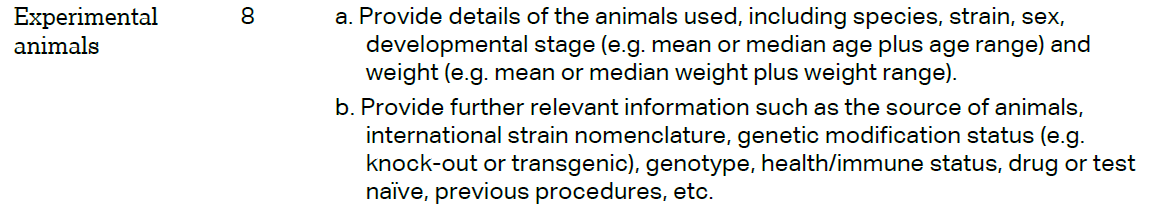 | | | Materials and Methods |

The ARRIVE guidelines. Originally published in *PLoS Biology*, June 2010^1^

| 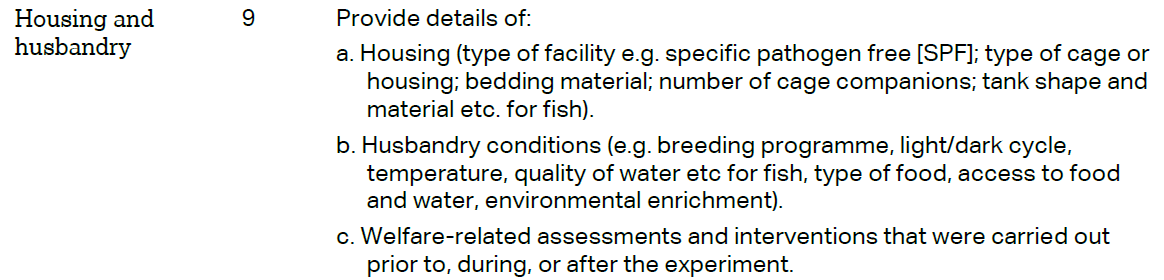 | Materials and Methods | |
| --- | --- | --- |
| 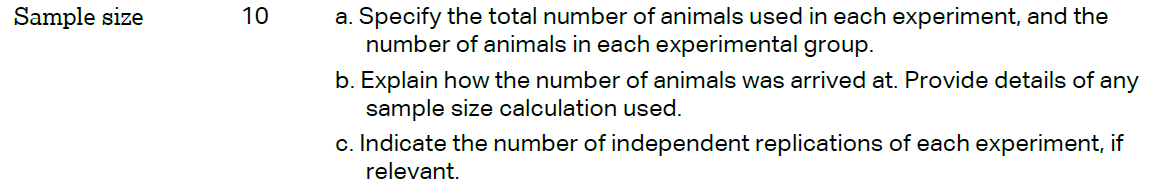 | Materials and Methods  Comply with the OECD guidelines (OECD 425) | |
| 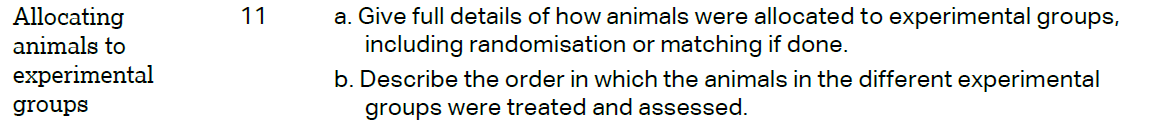 | Materials and Methods  Comply with the OECD guidelines (OECD 425) | |
| 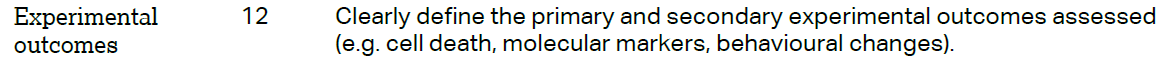 | Results and Discussion | |
| 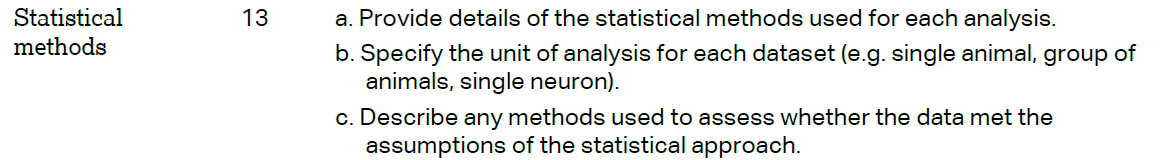 | Materials and Methods | |
| RESULTS |  | |
| 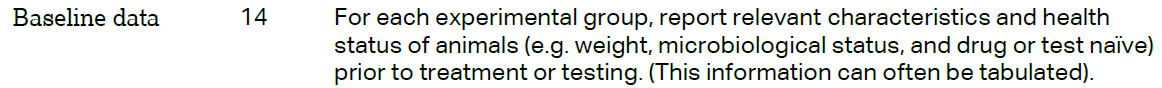 | See the attachment | |
| 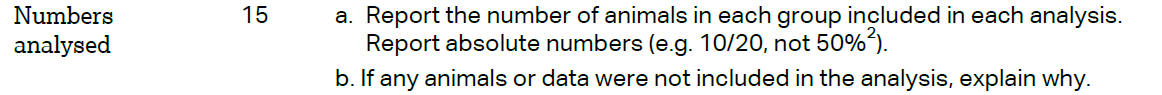 | See the attachment | |
| 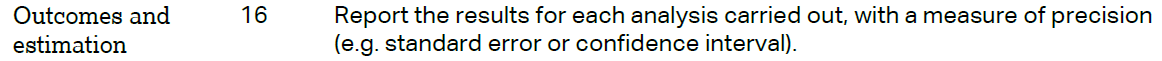 | Results and Discussion, Figures and Tables | |
| 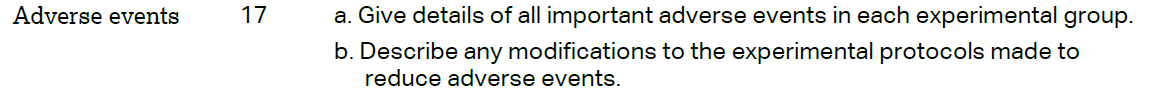 | See the attachment | |
| DISCUSSION |  | |
| 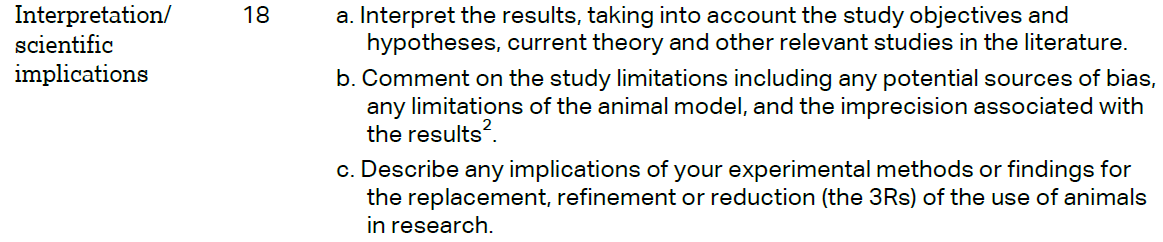 | See the attachment | |
| 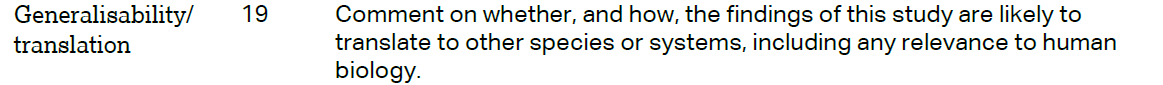 | See the attachment | |
| 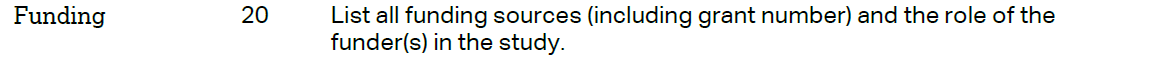 | See the attachment |  |


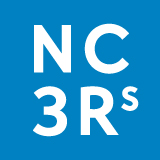

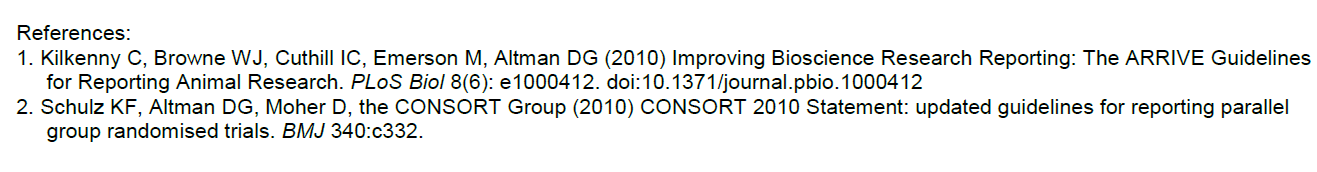


Item 1: Membrane-active Macromolecules Kill Antibiotic-tolerant Bacteria and Potentiate Antibiotics towards Gram-negative Bacteria

**Item 2:** The study aimed at finding a treatment of Gram-negative bacterial infections caused by antibiotic-resistant pathogens. The combination of membrane-active macromolecules and antibiotics was proposed for the treatment. First, the acute toxicity of molecules alone and in combination with antibiotics was tested in Female Balb/c mice (6-8 weeks, 18-22 g) using single-dose acute toxicity studies with different routes of administration (intravenous (i.v.) (tail vein), intraperitoneal (i.p.) and subcutaneous (s.c.) (over the flank)) studies. It was found that molecules alone and in combination with the antibiotic were found to be safe for performing the studies in mice. Next, antibacterial efficacy studies were performed in mice using burn wound and surgical infection models. The study showed that combination with antibiotics had significantly decreased bacterial burden compared to the untreated mice.

**Item 3: a)** Bacterial infections caused by multi-drug resistant Gram-negative bacteria have become a major threat to the global public health. The study aimed at providing a combination approach of membrane-active molecules and antibiotics for the treatment of Gram-negative bacterial infections including their biofilms. Towards this, first, the toxicity of the test drugs needed to be performed in mice to determine the maximum tolerable dose. Then, the antibacterial efficacy studies were performed in mice for determining the efficacy of the combination in-vivo.

**b)** Mice models can more or less be used to understand the bacterial infections and can be extended to humans. In the present study, we used routes of administration in mice like intravenous, intraperitoneal and subcutaneous that can also be performed in humans for administering the drugs. Also, the mouse burn wound and surgical infection models are generally used to mimic a tissue infection caused by bacteria in humans.

**Item 4:**  The primary objective of the study was to find a treatment option for Gram-negative burn wound bacterial infections caused by *A. baumannii* and biofilms. The secondary objective was to find out whether the combination of the molecules and antibiotics would be more efficacious than the individual molecules or antibiotics alone using the mice models.

**Item 5:** Animal studies were performed according to the protocols approved by Institutional Animal Ethics Committee (IAEC) of JNCASR and ICAR-NIVEDI. Toxicity studies were performed at JNCASR, Bengaluru (CPCSEA/201) and infection studies were performed at NIVEDI approved by the IAEC of NIVEDI, Bengaluru (881/GO/ac/05/CPCSEA) and the Catholic University of Brasilia (number 005/13).

**Item 6: a)** For the systemic toxicity studies 10 experimental groups and 3 control groups were used. For the infection studies, thirteen experimental groups and three control groups were used.

**b)** Animals were randomly selected, marked to permit individual identification and kept in their cages for at least 5 days before the experiment to allow for acclimatization to the experimental conditions.

**c)** The experiments were performed in individually ventilated cages (IVC) maintained with controlled environment as per the standards


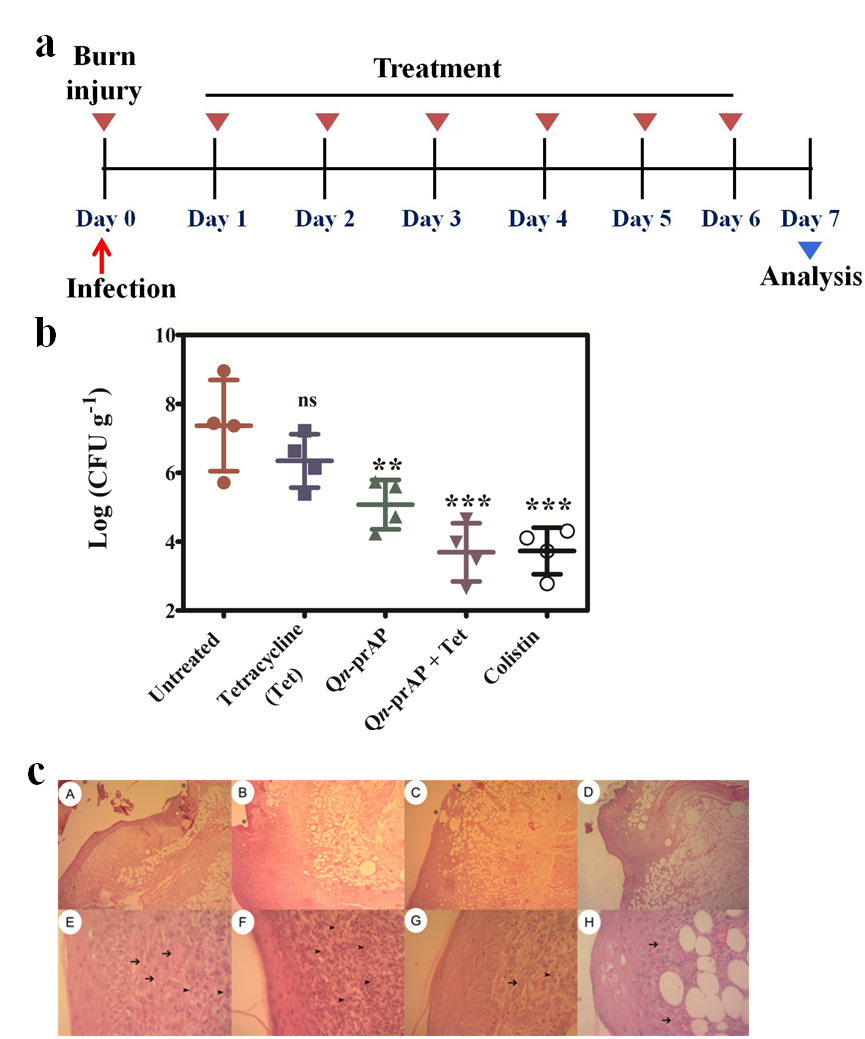


**Item 7-11: Pease see** Materials and Methods section of the manuscript. All the procedures comply with the OECD guidelines (OECD 425).

**Item 12:** Please see Results and discussion section of the manuscript.

**Item 13:** Please see Materials and Methods section of the manuscript.

**Item 14:** All the mice in the experimental groups were of the age of 6-8 weeks with an average body weight of 18-22 g prior to treatment or testing

**Item 15:** All the animals in each group were included in each analysis.

**Item 16:** Please see Results and discussion section of the manuscript.

**Item 17: a)** The mice in the high dose group (175 mg kg^-1^) in systemic toxicity studies immediately post-injection of the drug showed clinical signs of tremors, recumbency, sever distress and convulsions, which were indicative of the impending death or moribund condition.

**b)** For the intraperitoneal (i.p.) and subcutaneous (s.c.) (over the flank) routes of administration, the high dose (175 mg kg^-1^) was not injected to reduce the animal lethality.

**Item 18: a)** Please see Results and discussion section of the manuscript.

**Item 19:** The approach of using combination of two drugs for the treatment of bacterial infections was tested in mice models and can very well be translated to other species. As infections are universal, it can be translated in veterinary medicine for other animals and as well as in human medicine.

**Item 20:** The funders had no role in study design, data collection and analysis, decision to publish, or preparation of the manuscript.
