# Supplementary figures and images for: Membrane-active macromolecules kill antibiotic-tolerant bacteria and potentiate antibiotics towards Gram-negative bacteria
Source: PLoS One. 2017 Aug 24;12(8):e0183263. doi: 10.1371/journal.pone.0183263 (PMC5570306; doi:10.1371/journal.pone.0183263)

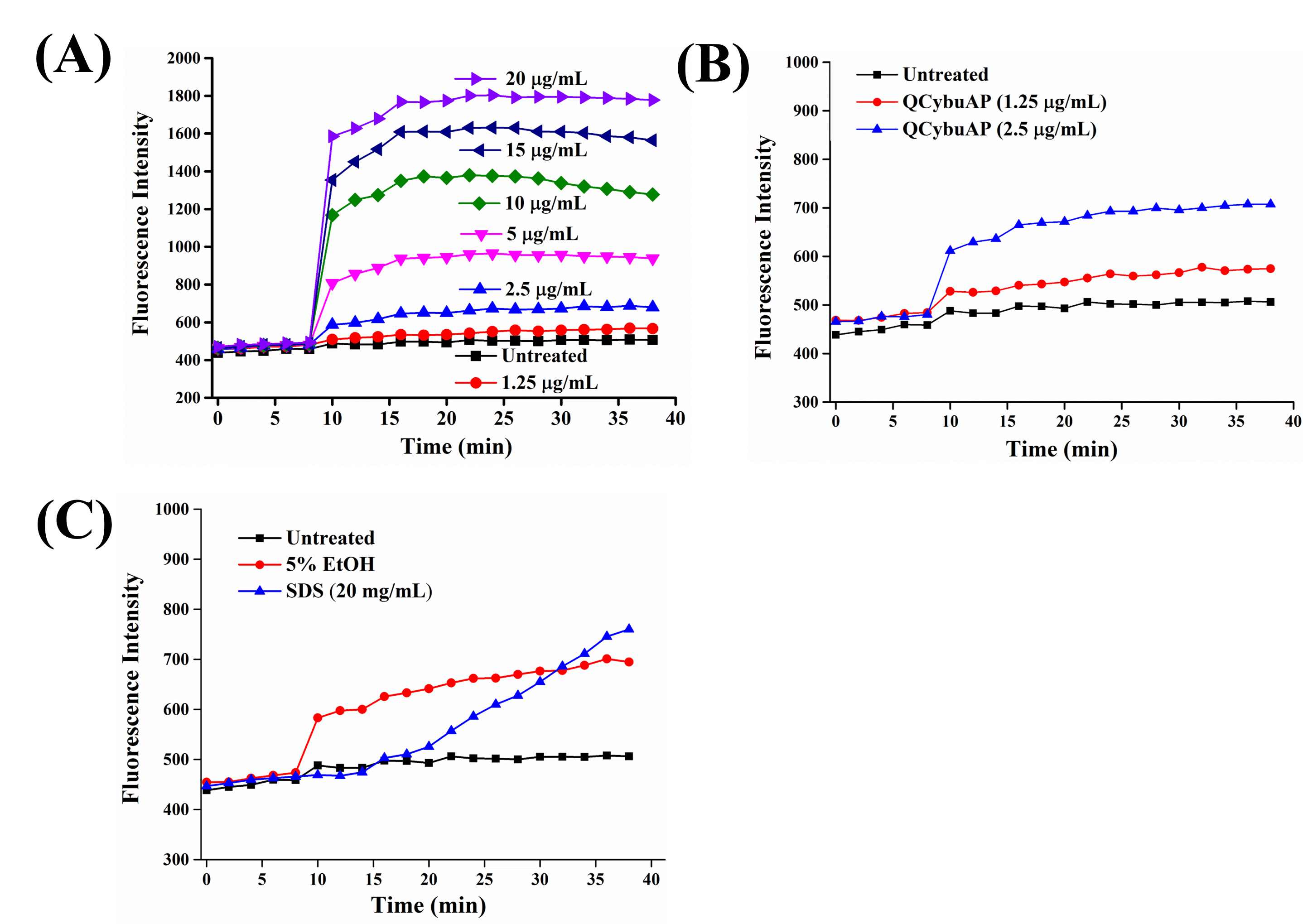

Supplement: S2 Fig — Concentration dependent effect of QCybuAP (A) and (B) Qn-prAP and also positive controls (C). (TIF) [file pone.0183263.s003.tif]

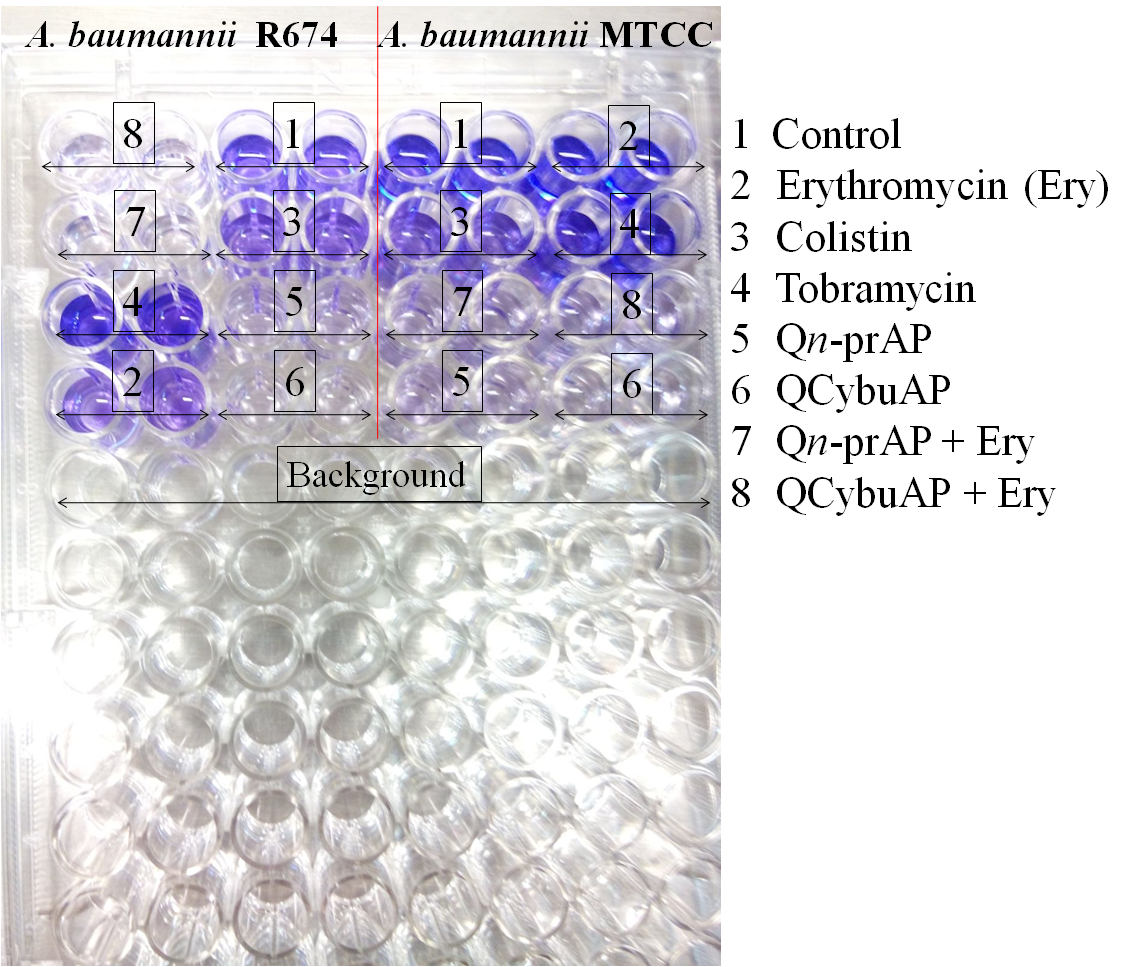

Supplement: S3 Fig — Biofilms grown on glass cover slips were treated in presence of colistin (30 μg mL-1), Qn-prAP and QCybuAP (both at 30 μg mL-1), erythromycin (Ery, 30 μg mL-1), tobramycin (Tobra, 30 μg mL-1), erythromycin + Qn-prAP/QCybuAP (30 μg mL-1 + 30 μg mL-1) or left untreated for 24 h. Crystal violet staining of the glass cover slips was performed and the dye was dissolved in 95% ethanol. The solution was transferred to a fresh 96-well plate and the image of the plate was taken with a digital camera. (TIF) [file pone.0183263.s004.tif]

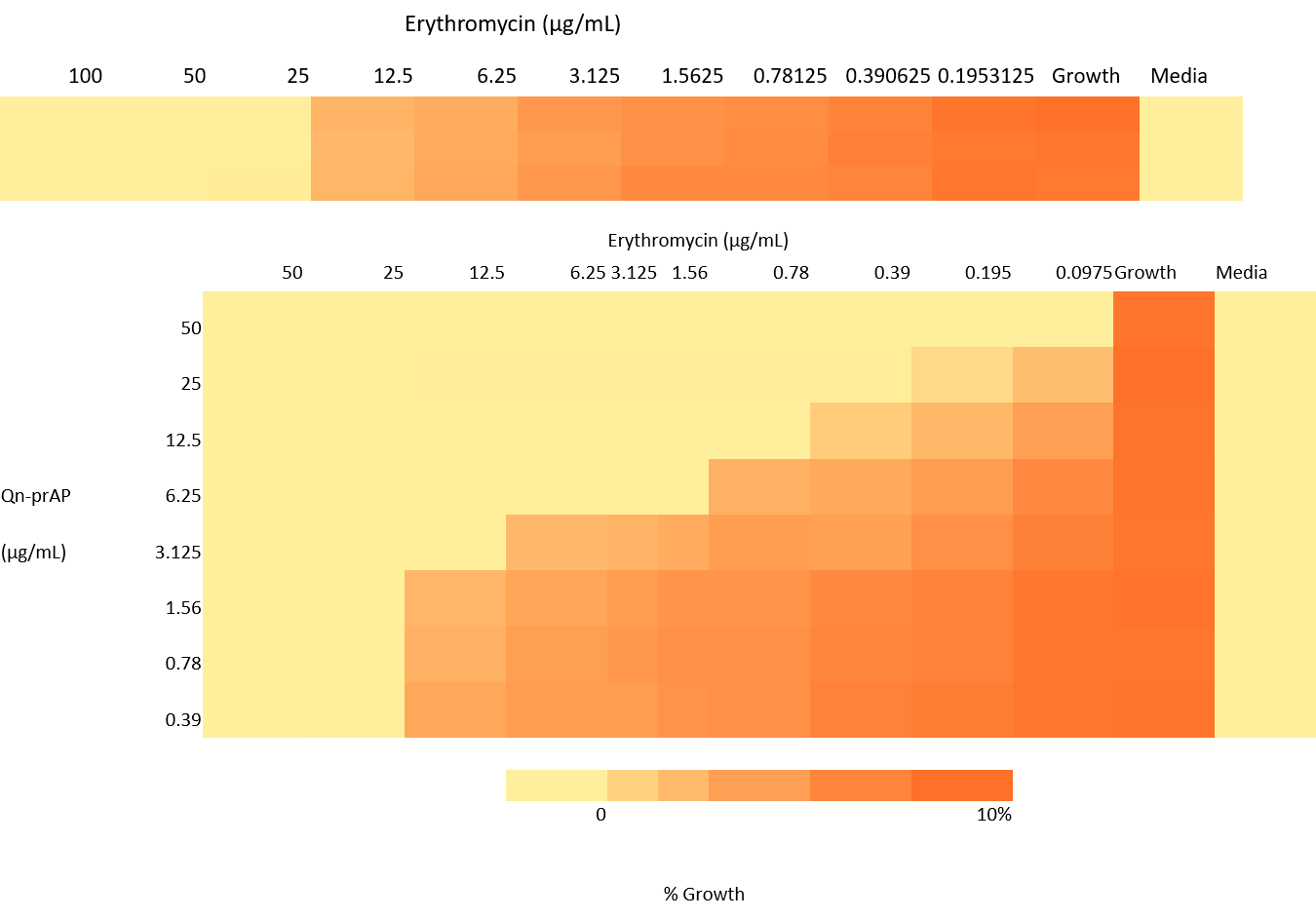

Supplement: S4 Fig — Antibacterial activity of erythromycin and Qn-prAP in combination with erythromycin against E. coli. (TIF) [file pone.0183263.s005.tif]

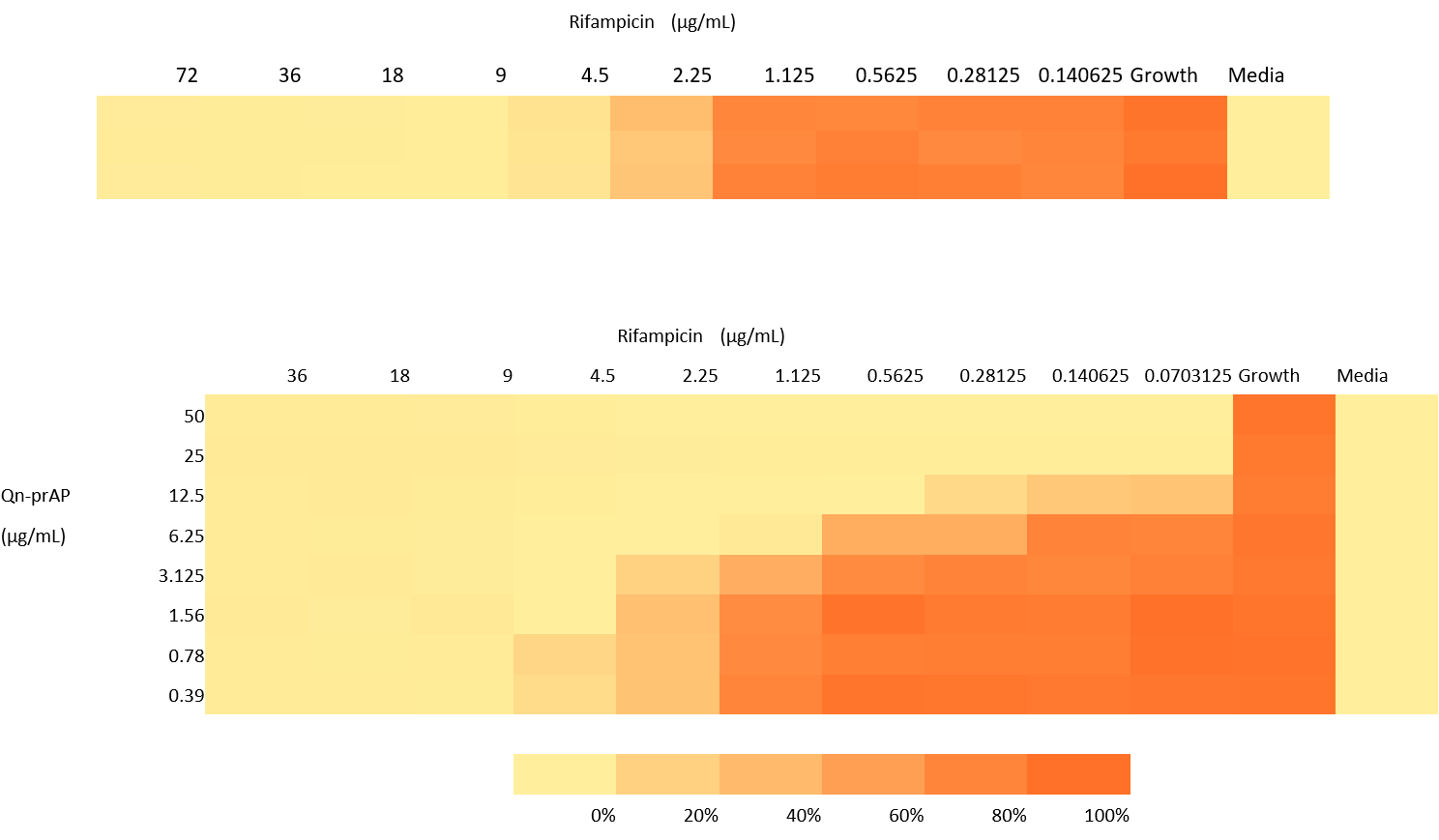

Supplement: S5 Fig — Antibacterial activity of Rifampicin and Qn-prAP in combination with rifampicin against E. coli. (TIF) [file pone.0183263.s006.tif]

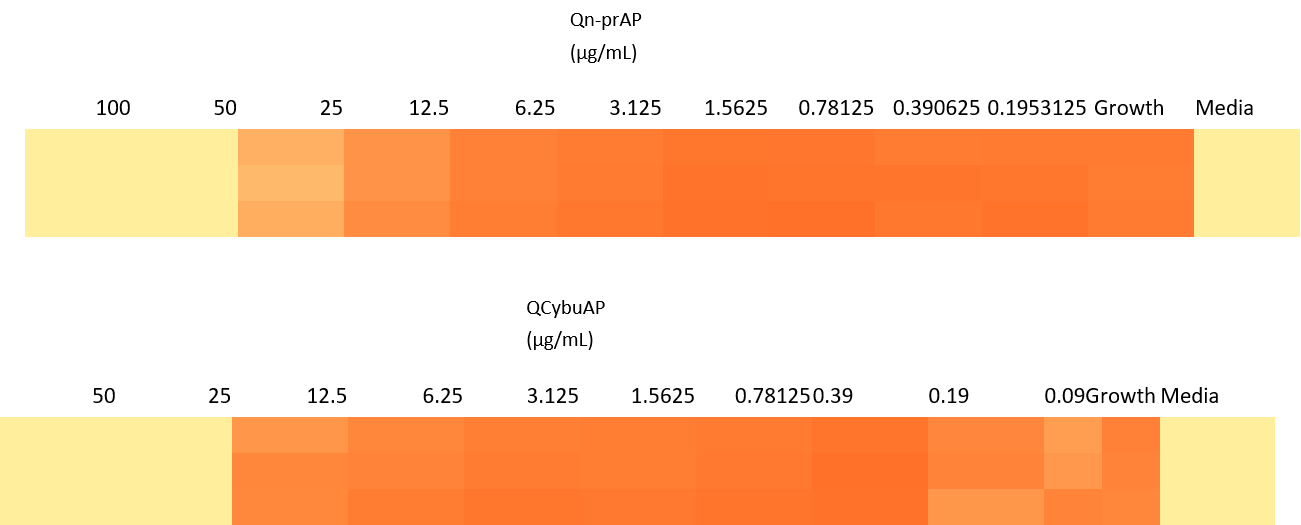

Supplement: S6 Fig — Antibacterial activity of Qn-prAP and QCybyAP against E. coli. (TIF) [file pone.0183263.s007.tif]

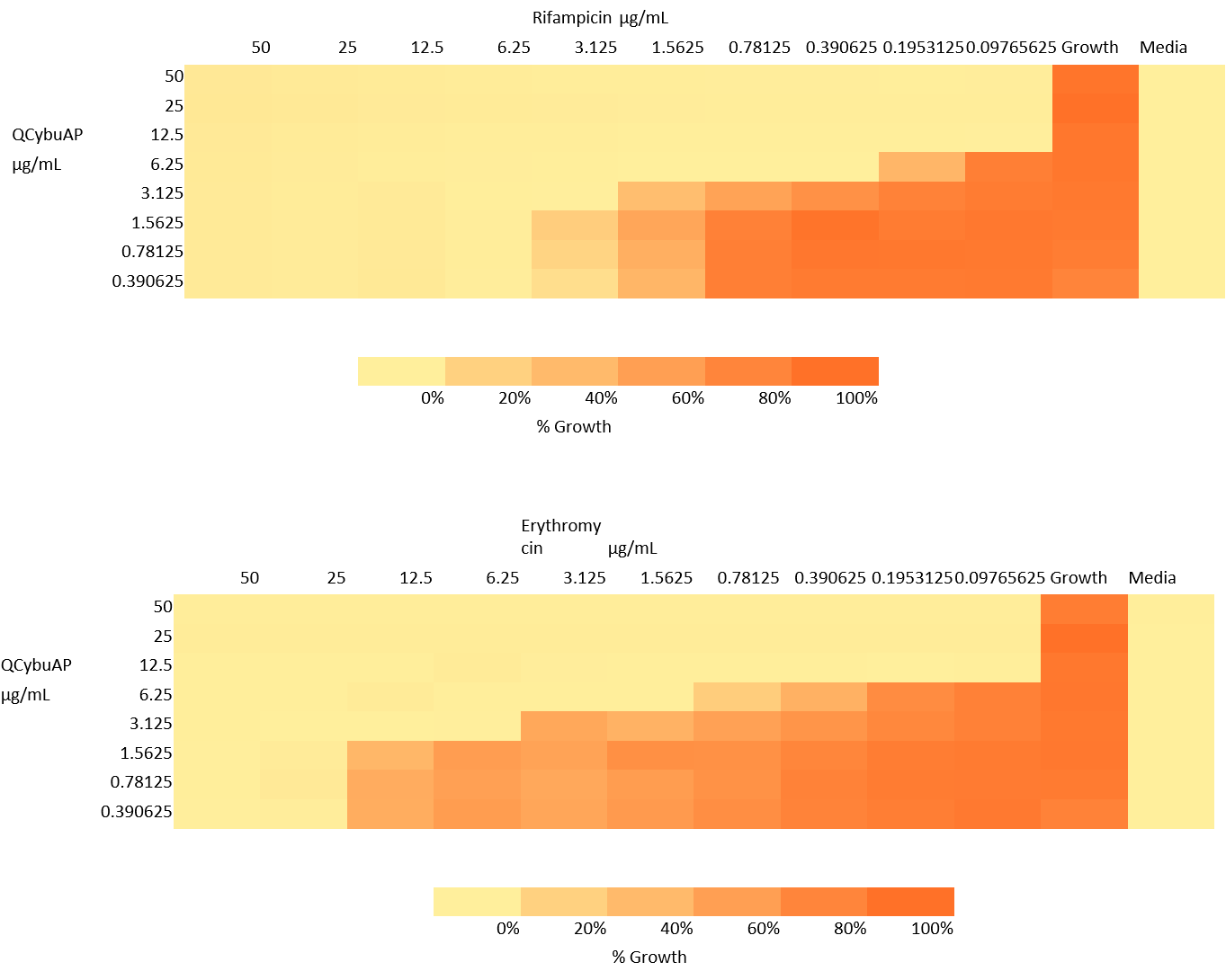

Supplement: S7 Fig — Antibacterial activity of QCybuAP in combination with erythromycin and rifampicin against E. coli. (TIF) [file pone.0183263.s008.tif]

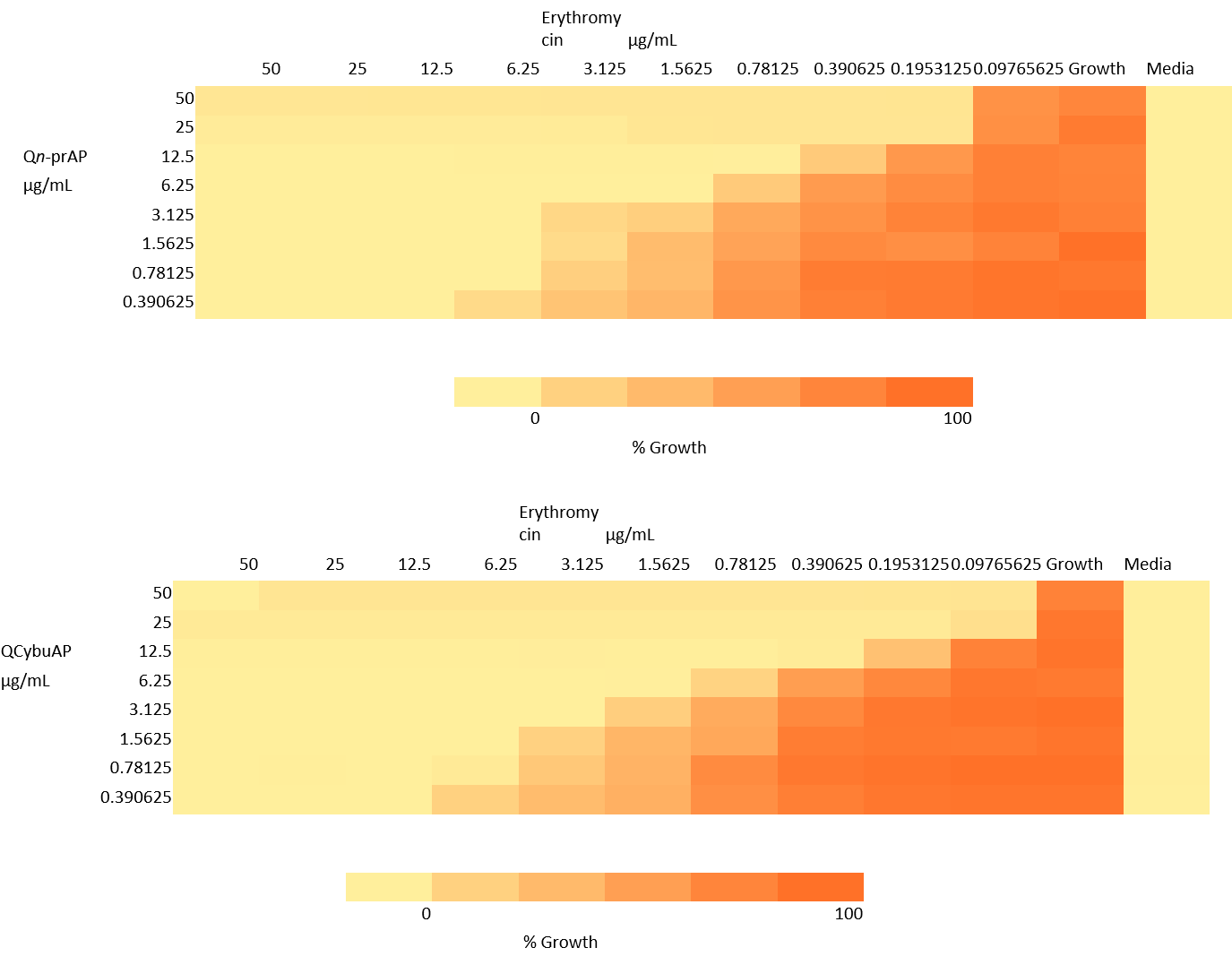

Supplement: S8 Fig — Antibacterial activity of QCybuAP and Qn-prAP in combination with erythromycin against A. baumannii. (TIF) [file pone.0183263.s009.tif]

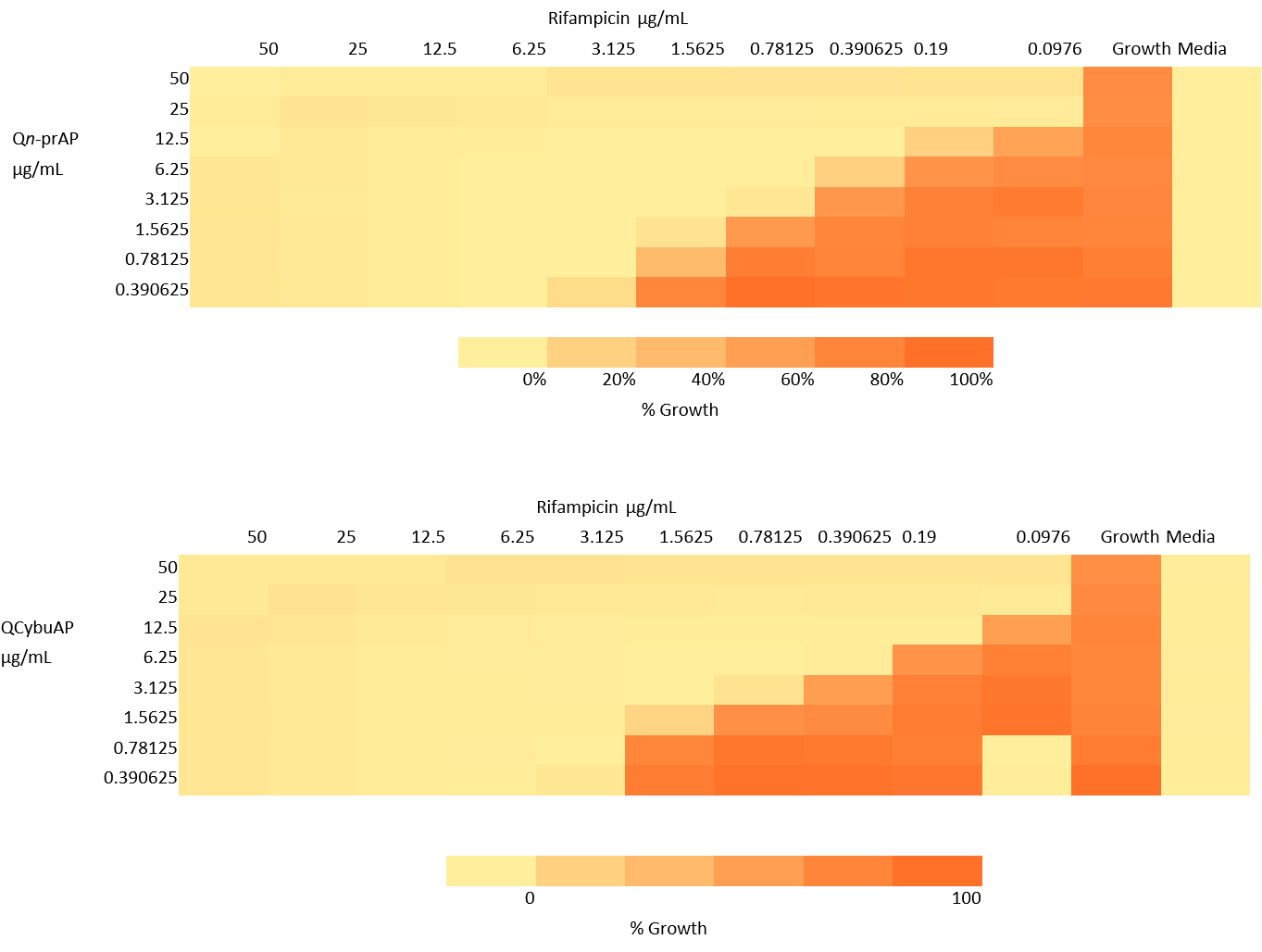

Supplement: S9 Fig — Antibacterial activity of QCybuAP and Qn-prAP in combination with rifampicin against A. baumannii. (TIF) [file pone.0183263.s010.tif]

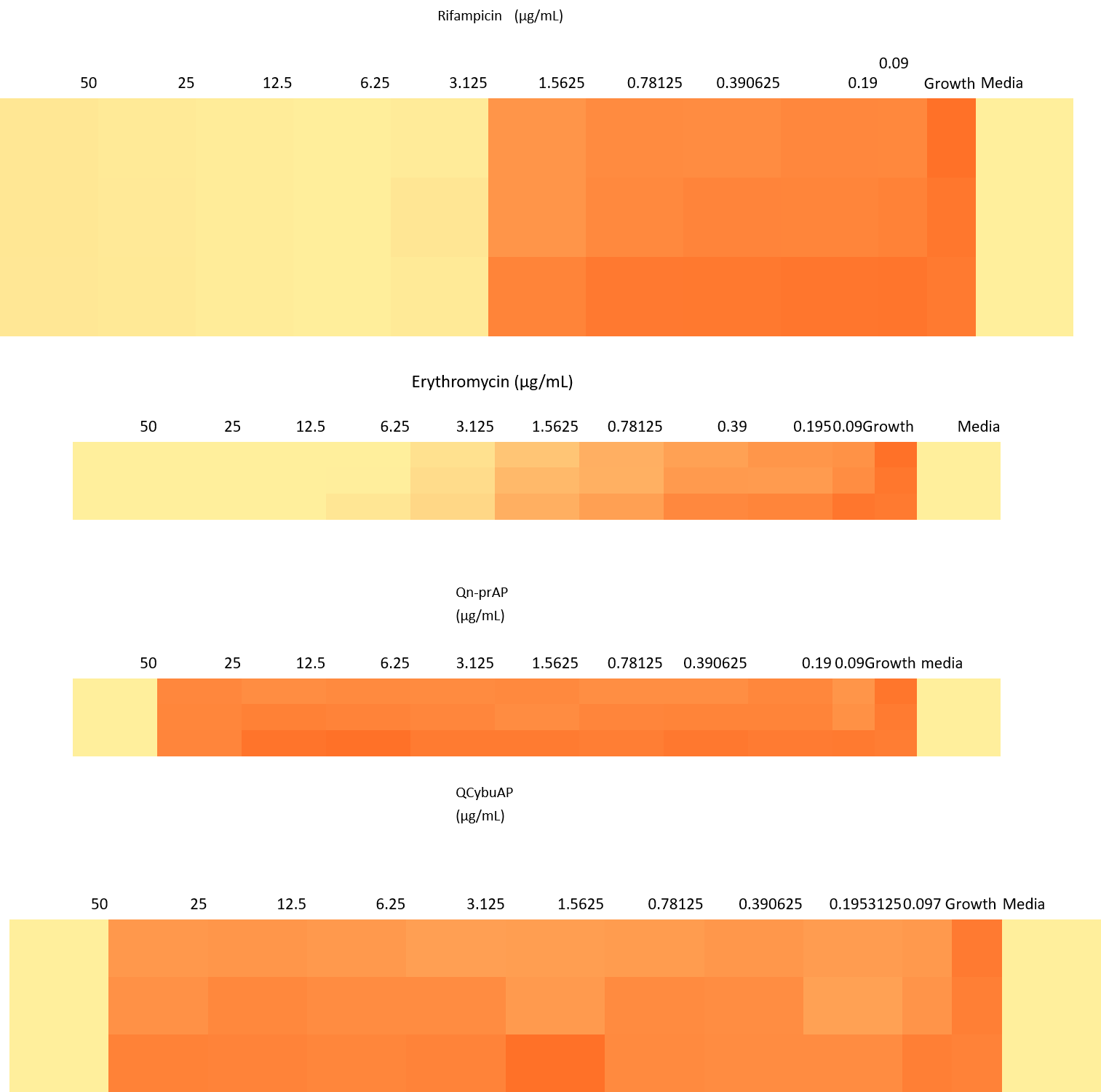

Supplement: S10 Fig — Antibacterial activity of QCybuAP, Qn-prAP, rifampicin and erythromycin against A. baumannii. (TIF) [file pone.0183263.s011.tif]

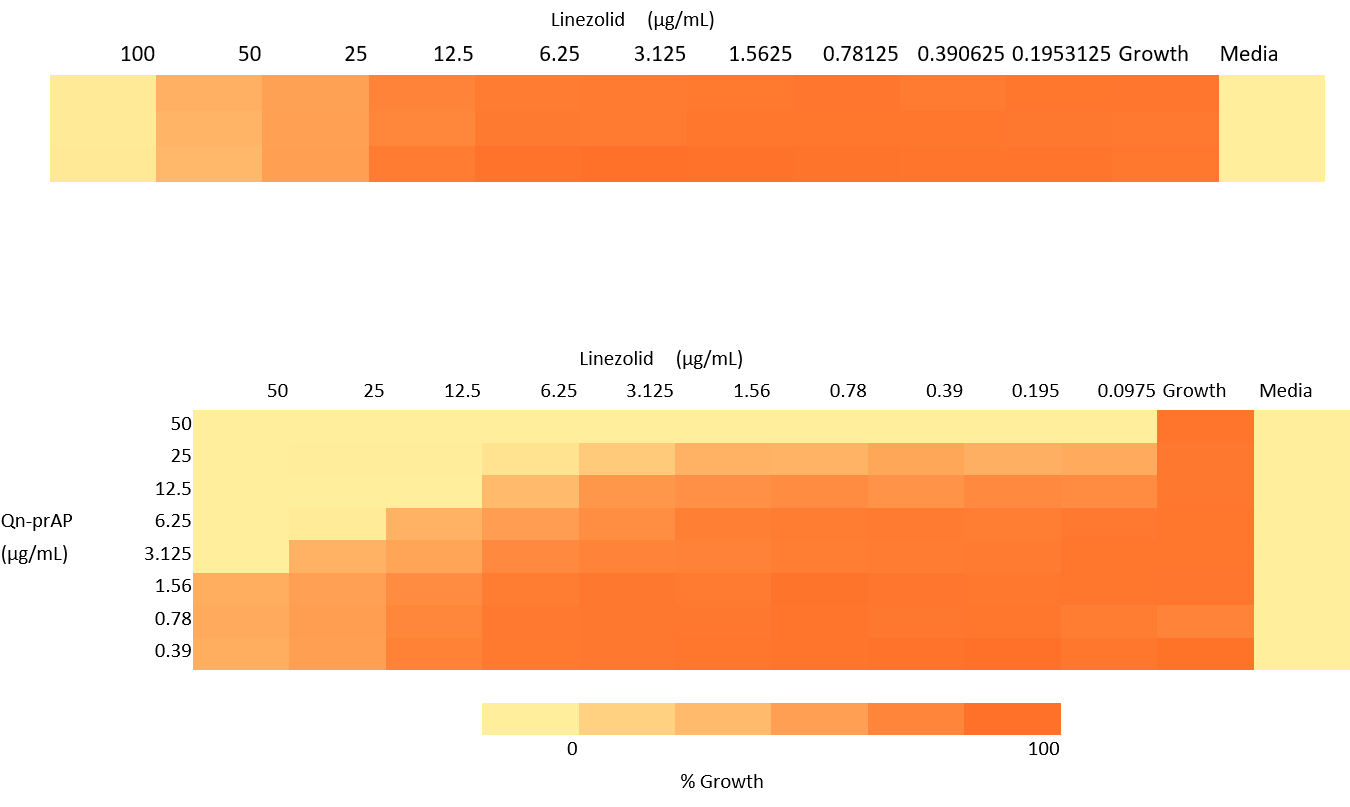

Supplement: S11 Fig — Antibacterial activity of in linezolid and Qn-prAP combination with Linezolid against E. coli. (TIF) [file pone.0183263.s012.tif]

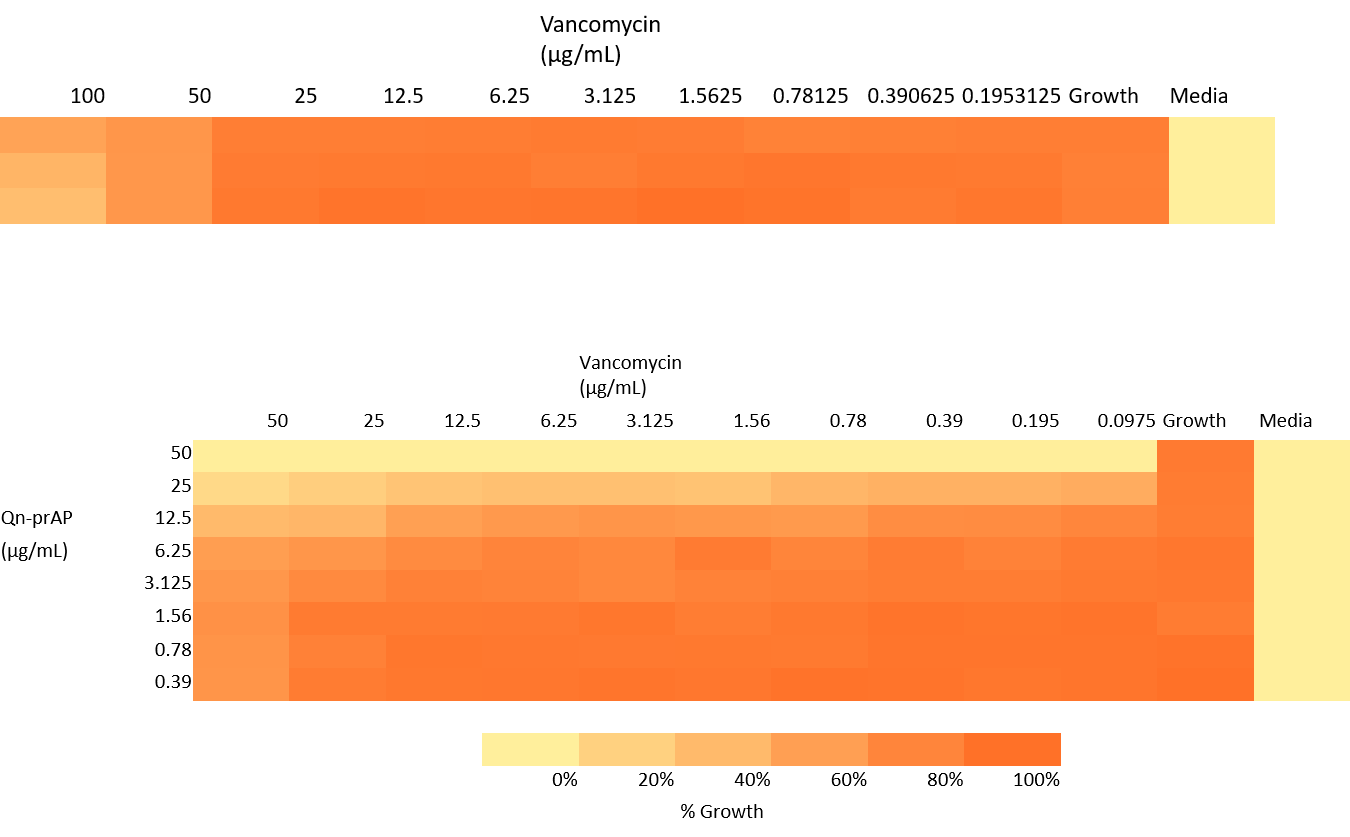

Supplement: S12 Fig — Antibacterial activity of in vancomycin and Qn-prAP combination with vancomycin against E. coli. (TIF) [file pone.0183263.s013.tif]

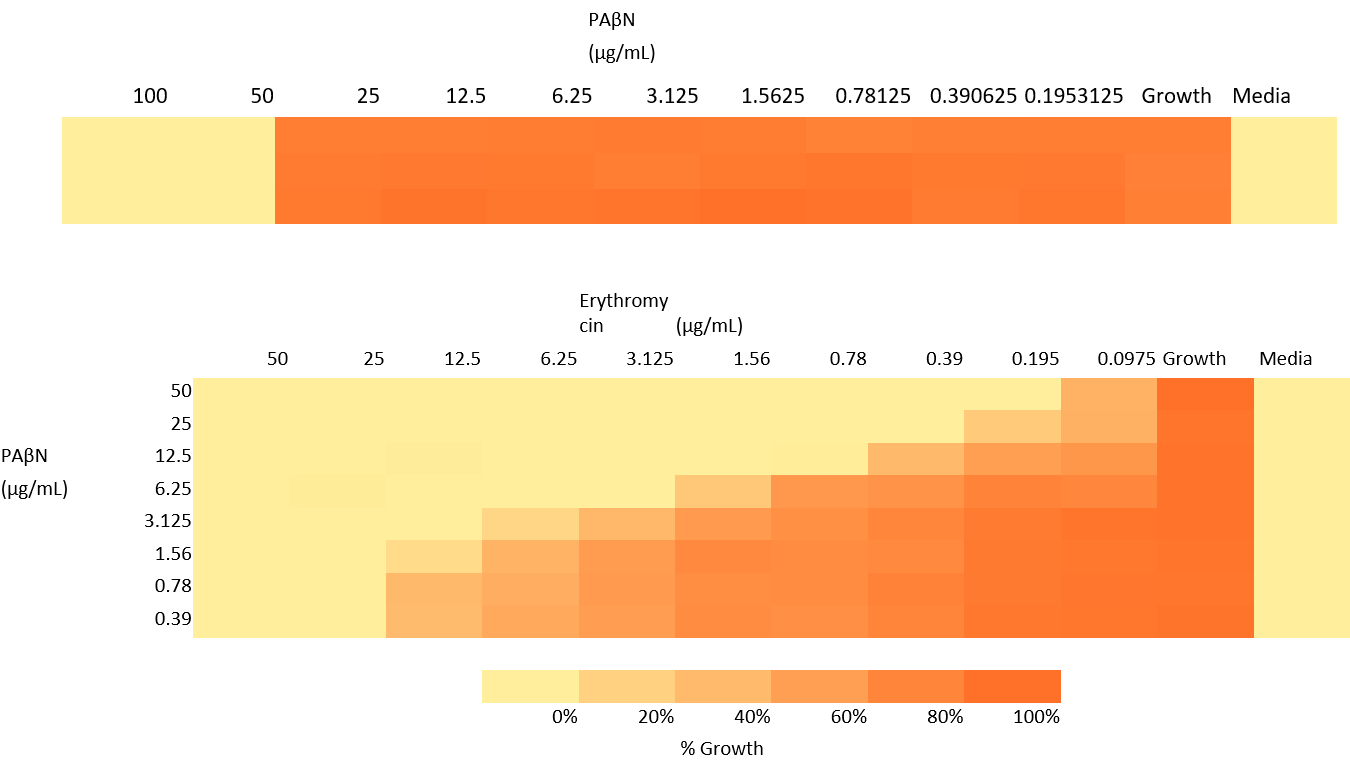

Supplement: S13 Fig — Antibacterial activity of PAβN and PAβN in combination with erythromycin against E. coli. (TIF) [file pone.0183263.s014.tif]

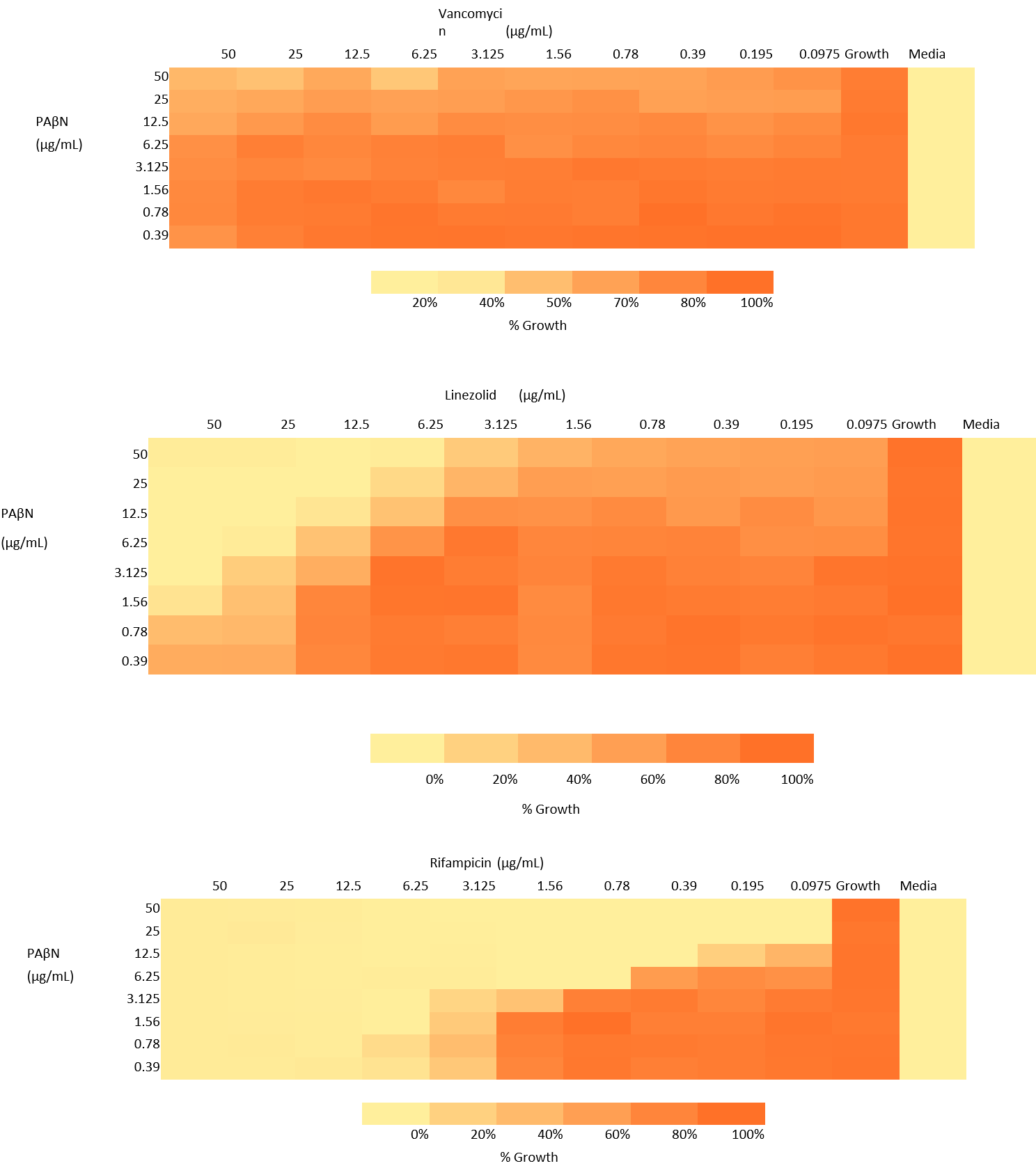

Supplement: S14 Fig — Antibacterial activity of PAβN in combination with rifampicin, linezolid and vancomycin against E. coli. (TIF) [file pone.0183263.s015.tif]

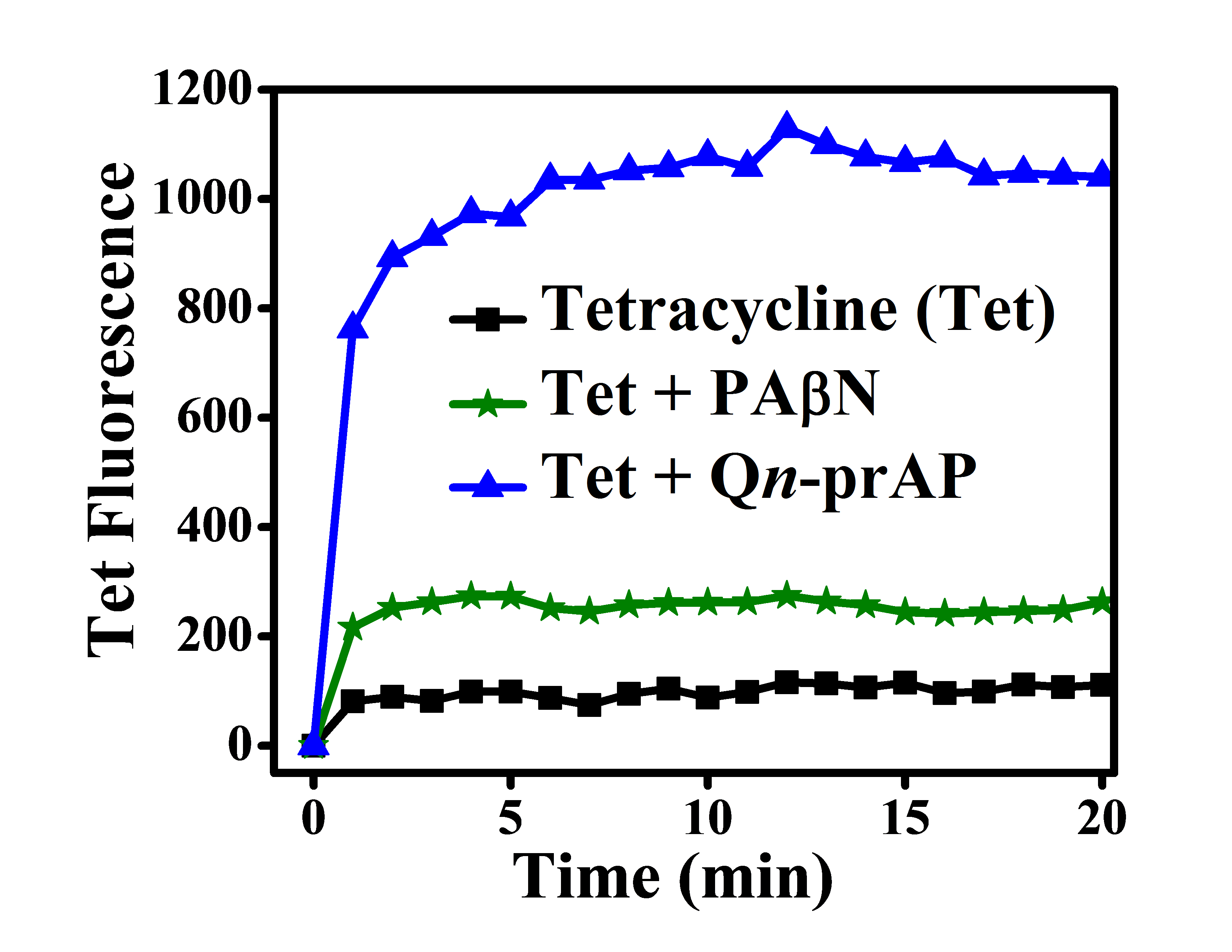

Supplement: S15 Fig — Uptake of tetracycline by increase in its fluorescence in presence of Qn-prAP (20 μg mL-1) and PAβN (50 μg mL-1). Tetracycline was used at 100 μg mL-1. Relative fluorescence was calculated by subtracting the fluorescence without the bacteria from the fluorescence of bacteria containing samples. (TIF) [file pone.0183263.s016.tif]

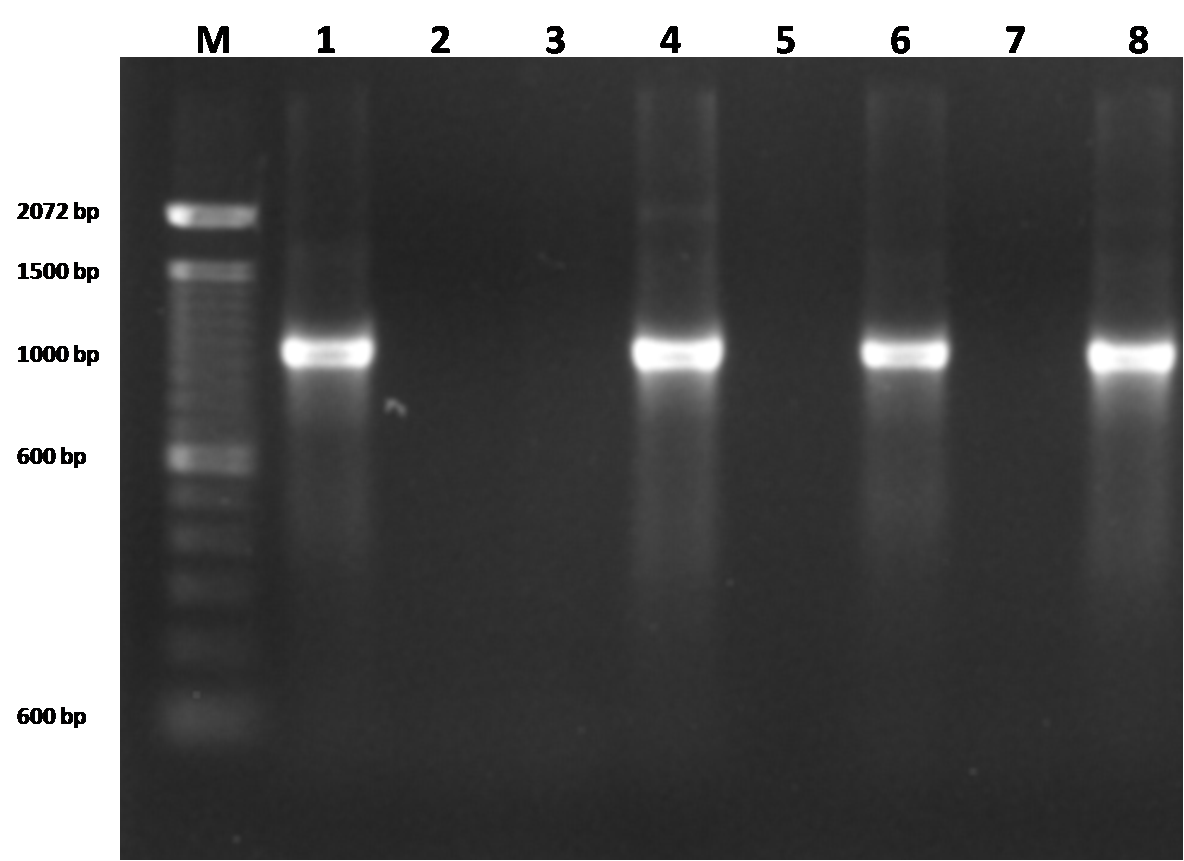

Supplement: S16 Fig — M-100 bp DNA ladder, Lane 1, positive control—K. pneumoniae IOC4955; Lane 2, negative control—K. pneumoniae ATCC700603; Lane 3, Negative clinical isolate—1; Lane 4, 003259271—K. pneumoniae; Lane 5, Negative clinical isolate– 2; Lane 6, Positive clinical isolate—1; Lane 7, Negative clinical isolate– 3; Lane 8, Positive clinical isolate—2. (TIF) [file pone.0183263.s017.tif]
