# Supplementary material for: Membrane-active macromolecules kill antibiotic-tolerant bacteria and potentiate antibiotics towards Gram-negative bacteria
Source: PLoS One. 2017 Aug 24;12(8):e0183263. doi: 10.1371/journal.pone.0183263 (PMC5570306; doi:10.1371/journal.pone.0183263)
Supplement: S1 Table — (DOCX) [file pone.0183263.s018.docx]

**S1 Table.** Antibiotic susceptibility data of *A. baumannii* R674 clinical isolate.

| **Antibiotic** | **MIC (µg mL^-1^)** | **Susceptibility** |
| --- | --- | --- |
| Ampicillin | >200 | R |
| Erythromycin | 200 | R |
| Tetracycline | 50 | R |
| Chloramphenicol | 200 | R |
| Ciprofloxacin | 200 | R |
| Kanamycin | 200 | R |
| Meropenem | 16-32 | R |
| Colistin | 0.5-1 | S |
| Rifampicin | >200 | R |

R- resistant.
