# Supplementary material for: Membrane-active macromolecules kill antibiotic-tolerant bacteria and potentiate antibiotics towards Gram-negative bacteria
Source: PLoS One. 2017 Aug 24;12(8):e0183263. doi: 10.1371/journal.pone.0183263 (PMC5570306; doi:10.1371/journal.pone.0183263)
Supplement: S2 Table — (DOCX) [file pone.0183263.s019.docx]

**S2 Table.** Antibacterial efficacy of polymers in combination with antibiotics.

| **Antibiotics and**  **bacterial strains** | **MIC of antibiotic (µg mL^-1^)** | | | | |  |
| --- | --- | --- | --- | --- | --- | --- |
|  | **-Polymers** | **+QCybuAP**  (µg mL^-1^) | | **+Q*n*-prAP**  (µg mL^-1^) | |  |
|  |  | 6.25 | 12.5 | 6.25 | 12.5 |  |
| *E. coli* |  |  |  |  |  |  |
| Erythromycin | 38 ± 18 | 1.6(0.29) | 0.1(0.5) | 1.6(0.17) | 0.8(0.27) |  |
| Rifampicin | 7.6 ± 1.9 | 0.4(0.3) | 0.1(0.51) | 1.35 ± 0.35(0.32) | 0.4 ±(0.27) |  |
|  |  |  |  |  |  |  |
| *A. baumannii* |  |  |  |  |  |  |
| Erythromycin | 6.2 | 1.5(0.37) | 0.4(0.31) | 1.5(0.37) | 0.8(0.38) |  |
| Rifampicin | 3.1 | 0.4(0.25) | 0.2(0.31) | 0.8(0.38) | 0.4(0.38) |  |
|  |  |  |  |  |  |  |

Values in the parenthesis indicate FICI (fractional inhibitory concentration index) values.
