# Supplementary material for: Membrane-active macromolecules kill antibiotic-tolerant bacteria and potentiate antibiotics towards Gram-negative bacteria
Source: PLoS One. 2017 Aug 24;12(8):e0183263. doi: 10.1371/journal.pone.0183263 (PMC5570306; doi:10.1371/journal.pone.0183263)
Supplement: S3 Table — (DOCX) [file pone.0183263.s020.docx]

**S3 Table.** Antibacterial efficacy in nutrient broth.

| **Bacteria** | **MIC (µg mL^-1^)** | | | | | |  |
| --- | --- | --- | --- | --- | --- | --- | --- |
|  | **QCybuAP** | **Q*n*-prAP** | | **PAβN** | | |  |
| *E. coli* | 25 | 50 |  |  | 50 | |  |
|  |  |  |  |  |  | |  |
| *A. baumannii* | 50 | 50 |  |  | 50 | |  |
|  |  |  |  |  |  |  |  |
