# Supplementary material for: Membrane-active macromolecules kill antibiotic-tolerant bacteria and potentiate antibiotics towards Gram-negative bacteria
Source: PLoS One. 2017 Aug 24;12(8):e0183263. doi: 10.1371/journal.pone.0183263 (PMC5570306; doi:10.1371/journal.pone.0183263)
Supplement: S4 Table — (DOCX) [file pone.0183263.s021.docx]

**S4 Table.** Role of efflux pumps in antibiotic resensitization towards Gram-negative bacteria.

| **Antibiotics and**  **bacterial strain** | **MIC of antibiotic (µg mL^-1^)** | | | | | |  |
| --- | --- | --- | --- | --- | --- | --- | --- |
|  | **-Polymers** | **+Q*n*-prAP**  (µg mL^-1^) | | **+PAβN^a^**  (µg mL^-1^) | | |  |
|  |  | 6.25 | 12.5 | 12.5 | 25 | |  |
| *E. coli* |  |  |  |  |  | |  |
| Erythromycin | 38 ± 18 | 1.6(0.17) | 0.8(0.27) | 0.8(0.27) | 0.4(0.51) | |  |
| Rifampicin | 7.6 ± 1.9 | 1.35 ± 0.35(0.32) | 0.4 ± 0.28(0.27) | 0.4(0.3) | 0.1(0.51) | |  |
| Linezolid | 100 | 25(0.375) | 12.5(0.38) | 25(0.5) | 12.5(0.63) | |  |
| Vancomycin | >100 | >100(N.D.) | >100(N.D.) | >100(N.D.) | >100(N.D.) |  |  |

^a^PAβN- phenyl arginine β-naphthylamide. Values in the parenthesis indicate FICI values. N.D.- not determined.
