# Supplementary material for: Membrane-active macromolecules kill antibiotic-tolerant bacteria and potentiate antibiotics towards Gram-negative bacteria
Source: PLoS One. 2017 Aug 24;12(8):e0183263. doi: 10.1371/journal.pone.0183263 (PMC5570306; doi:10.1371/journal.pone.0183263)
Supplement: S5 Table — (DOCX) [file pone.0183263.s022.docx]

**S5 Table.** Antibiotic susceptibility data of *K. pneumoniae-*003259271 (carbapenemase producing strain, KPC) clinical isolate.

| **Antibiotic** | **MIC (µg mL^-1^)** | **Susceptibility** |
| --- | --- | --- |
| Amikacin MIC | ≤16 | S |
| Ampicillin/Sulbactam | >16/8 | R |
| Ampicillin | >16 | R |
| Aztreonam | >16 | R |
| Cefepime | >16 | R |
| Cefotaxime | >32 | R |
| Cefotaxime/K Clavulanate | >4 | R |
| Cefotetan | >32 | R |
| Cefoxitin | NT | NT |
| Ceftazidime | >16 | R |
| Ceftazidime/K Clavulanate | >2 | R |
| Ceftriaxone | >32 | R |
| Cefuroxime | >16 | R |
| Cephalothin | >16 | R |
| Ciprofloxacin | >2 | R |
| Ertapenem | >4 | R |
| Gemifloxacin | NT | NT |
| Gentamicin | ≤4 | S |
| Imipenem | >8 | R |
| Levofloxacin | >4 | R |
| Meropenem | >8 | R |
| Nitrofurantoin | >64 | R |
| Pipericillin/Tazobactam | >64 | R |
| Piperacillin | >64 | R |
| Tetracycline | >8 | R |
| Tigecycline | ≤2 | S |
| Tobramycin | >8 | R |
| Trimethoprim/  Sulfamethoxazole | >2/38 | R |

S- sensitive, R- resistant, NT- not tested.
